# Microbial synthesis of titanium dioxide nanoparticles and their importance in wastewater treatment and antimicrobial activities: a review

**DOI:** 10.3389/fmicb.2023.1270245

**Published:** 2023-10-16

**Authors:** Chandani Rathore, Virendra Kumar Yadav, Amel Gacem, Siham K. AbdelRahim, Rakesh Kumar Verma, Rajendra Singh Chundawat, G. Gnanamoorthy, Krishna Kumar Yadav, Nisha Choudhary, Dipak Kumar Sahoo, Ashish Patel

**Affiliations:** ^1^Department of Biosciences, School of Liberal Arts and Sciences, Mody University of Science and Technology, Laxmangarh, Rajasthan, India; ^2^Department of Life Sciences, Hemchandracharya North Gujarat University, Patan, Gujarat, India; ^3^Department of Physics, Faculty of Sciences, University 20 Août 1955, Skikda, Algeria; ^4^Department of Chemistry, College of Science, King Khalid University, Abha, Saudi Arabia; ^5^Department of Inorganic Chemistry, University of Madras, Chennai, Tamilnadu, India; ^6^Faculty of Science and Technology, Madhyanchal Professional University, Ratibad, Bhopal, India; ^7^Environmental and Atmospheric Sciences Research Group, Scientific Research Center, Al-Ayen University, Nasiriyah, Iraq; ^8^Department of Veterinary Clinical Sciences, College of Veterinary Medicine, Iowa State University, Ames, IA, United States

**Keywords:** titanium dioxide, photocatalytic degradation, dye removal, microbial synthesis, waste water

## Abstract

Nanotechnology (NT) and nanoparticles (NPs) have left a huge impact on every field of science today, but they have shown tremendous importance in the fields of cosmetics and environmental cleanup. NPs with photocatalytic effects have shown positive responses in wastewater treatment, cosmetics, and the biomedical field. The chemically synthesized TiO_2_ nanoparticles (TiO_2_ NPs) utilize hazardous chemicals to obtain the desired-shaped TiO_2_. So, microbial-based synthesis of TiO_2_ NPs has gained popularity due to its eco-friendly nature, biocompatibility, etc. Being NPs, TiO_2_ NPs have a high surface area-to-volume ratio in addition to their photocatalytic degradation nature. In the present review, the authors have emphasized the microbial (algae, bacterial, fungi, and virus-mediated) synthesis of TiO_2_ NPs. Furthermore, authors have exhibited the importance of TiO_2_ NPs in the food sector, automobile, aerospace, medical, and environmental cleanup.

## 1. Introduction

Nanotechnology and nanoscience have gained huge importance in the last few years due to their exceptional features (Ray and Bandyopadhyay, 2021; Modi et al., 2022b; Zanata et al., 2022). Nanoparticles (NPs) have gained attention in the fields of environmental cleanup, electronics, research, medicine, etc. (Singh Jassal et al., 2022). The increase in demand for NPs is mainly due to their high surface area-to-volume ratio (SVR) and high surface energy, which makes them a potential candidate for a wide range of applications (Egbosiuba et al., 2020). On the basis of elements, the NPs could be categorized into two types: one is metallic and the other is non-metallic (Yadav et al., 2020a; Amari et al., 2023). The metallic NPs include both metal oxides and metal NPs, whereas the metal NPs mainly include gold (Au), silver (Ag), Ti, platinum (Pt), copper (Cu), and Fe (0). The metal oxide NPs include titanium dioxide (TiO_2_) (Kiwi et al., 2014), zinc oxide (ZnO) (Modi et al., 2022a, 2023b; Onyszko et al., 2022), iron oxide (Fe_2_O_3_/Fe_3_O_4_) (Pan et al., 2022; Yadav et al., 2023a), magnesium oxide (MgO) (Dabhane et al., 2022), copper oxide (CuO) (Maliki et al., 2022), alumina (Al_2_O_3_), and many more (Ravichandran, 2010; Guerra et al., 2018). Among non-metallic ones, the most prominent are silica oxide (SiO_2_) (Huang et al., 2022; Imoisili and Jen, 2022; Imoisili et al., 2022; Yadav et al., 2023c), graphene, and carbon nanotubes (CNTs) (Guerra et al., 2018). Out of all the metal oxides, ZnO and TiO_2_ have gained a lot of attention in recent years due to their photocatalytic properties (Wang et al., 2021a; Zhao et al., 2022). In comparison with other metal oxides, TiO_2_ NPs are a better choice for several applications due to their photocatalytic nature, low cost, high abundance, self-cleaning activities, strong oxidizing power, and better chemical stability (Yadav et al., 2022b). It is an N-type semiconductor because of the presence of oxygen vacancies, which favor the development of positive electrons or Ti^3+^ centers and hence excess e^−^ donors in the electronic structure of titanium (Shi et al., 2022). The two major drawbacks of utilizing undoped TiO_2_ as a photocatalyst are its wide band gap of 3.00–3.30 eV (which depends on the polymorph of TiO_2_ used) and high charge carrier recombination rate (Zheng et al., 2020; Žerjav et al., 2022). So, this issue could be overcome by using a UV source as TiO_2_ exhibits photocatalytic behavior in the presence of a source of UV light.

Based on the crystallinity, TiO_2_ can be classified either as amorphous or crystalline (Chen et al., 2020a; Chakhtouna et al., 2021). Moreover, TiO_2_ could exist in three polymorphs, namely anatase, rutile, and brookite. Out of all these three polymorphs, anatase is most extensively exploited for photocatalytic applications due to its higher photocatalytic activity in comparison with anatase and brookite. Among all the three polymorphs, rutile has the narrowest band gap of ~3.0 eV but commonly expresses up to an order of magnitude lower photocatalytic activity than anatase. The utilization of pure brookite polymorphs in heterogeneous photocatalysis is a very challenging task due to their complex synthesis method, even though they could exhibit higher photocatalytic activity than the other two polymorphs. Moreover, the thermodynamic metastability of brookite is very low. Due to this reason, brookite is the least studied form of TiO_2_. There are several cases where mixed phases of TiO_2_ have been obtained and exhibited comparatively higher photocatalytic activity than the individual polymorphs (Žerjav et al., 2022). Rutile is the most stable crystalline form of TiO_2_, which forms at a temperature of ~800°C (Yadav et al., 2014a). The amorphous form of TiO_2_ (anatase) has irregular morphology due to the arrangement of the particles in a random fashion. This phase of TiO_2_ generally forms at ~350°C. In addition to this, the less stable anatase and brookite irreversibly get transformed into the stable rutile polymorpha at a temperature of ~500–800°C. Out of all the three polymorphs, anatase is the most photosensitive in comparison with rutile and brookite (Eddy et al., 2023).

TiO_2_ NPs can be synthesized by all three approaches, namely chemical, physical, and biological methods. The chemical method is quick and takes less time, but due to the utilization of more chemical agents, this approach is not eco-friendly. The various chemical approaches for the formation of TiO_2_ NPs are hydrothermal, sonochemical (Khan et al., 2016), thermal decomposition, chemical vapor deposition (CVD), and sol-gel techniques (Mir et al., 2017; Rajendran et al., 2021). The physical approach mainly includes the ball milling technique and physical vapor deposition (PVD) but is quite expensive and energy-intensive. Due to all these limitations, there is a need for the biological synthesis (plants and microbes) of TiO_2_ NPs due to their environment-friendly properties and biocompatibility for their application in the medical field. The biological method is the best method for the synthesis of TiO_2_ NPs (Aravind et al., 2021).

Among biological methods, the microbial approach is quite effective and efficient due to the shorter time taken by the microorganisms to grow in comparison with plants. Microorganisms have various biomolecules such as peptides, proteins, enzymes, lipids, and carbohydrates that can be used by microorganisms to transform metallic salts into their respective NPs (Phogat et al., 2018; Dhara and Nayak, 2022). Moreover, the biomolecules present in these microbes may play the role of a capping agent to get the NPs of uniform and desired morphology (Verma and Mehata, 2016). To date, several microorganisms such as *Bacillus subtilis* (bacteria)*, Staphylococcus aureus* (bacteria)*, Streptomyces* (actinomycetes)*, Aspergillus* sps. (fungi), and *Spirulina* sps. (algae) have been used by the investigators for the synthesis of TiO_2_ NPs (Singh Jassal et al., 2022; Verma et al., 2022). Steps involved in the biosynthesis of TiO_2_ NPs are the isolation of appropriate microbes, the addition of precursors to the bacterial culture, the characterization of NPs, and their applications. Microorganisms generally synthesize NPs by two approaches: either extracellular or intracellular (Yadav et al., 2020b). During the intracellular synthesis of NPs, first, the metal ions, including Ti^3+^ ions, get entrapped by the microorganisms, followed by the enzymatic reduction of the metallic ions within the cell wall as mentioned above (Alfryyan et al., 2022). In the extracellular mechanism (Kulkarni et al., 2023), the enzyme is secreted outside, where the metal ions get transformed into metal oxides outside the cell. In this study, the bioreduction process takes place, and the NPs are thereafter produced (Qamar and Ahmad, 2021). Hasanin et al. (2023) reported the synthesis of ZnO-CuO NPs/CSC by using *Aspergillus niger* AH1 and examined their photocatalytic activity. Fouda et al. (2021a) reported the synthesis of γ-Fe_2_O_3_-NPs by using *Penicillium expansum* strain (K-w) and applied them for the treatment of tannery and textile wastewater. Fouda et al. (2021b) also synthesized MgO-NPs by using the fungus *A. niger* F1 and utilized them for the removal of real textile and tannery effluent. Saied et al. (2022) synthesized hematite NPs by using the fungus *A. niger*, AH1, and further assessed their antimicrobial and photocatalytic activities.

In most of the bacterial-mediated synthesized TiO_2_ NPs, the investigators have used both Gram-positive and Gram-negative bacteria. In addition to these eukaryotic microorganisms, other eukaryotic microorganisms (fungi, yeast, mushrooms, and algae) have also been used for the synthesis of TiO_2_ NPs. In the majority of the cases, investigators have used bacterial culture supernatant for the biosynthesis of TiO_2_ NPs (Srinivasan et al., 2022; Rathi and Jeice, 2023). Moreover, the most preferred titanium precursors were titanyl sulfate and titanyl hydroxide, whose molarity was mainly 0.025 mM. In addition to this, some of them have also utilized micron-sized TiO_2_ as a precursor. In the majority of cases, the synthesis of TiO_2_ NPs involved the growth of bacterial culture, harvesting, centrifugation to obtain supernatant, mixing of titanium precursor and bacterial supernatant, heating for a few minutes to hours, and finally shaking in an incubator for 24–72 h. Most of the approaches have used TiO_2_ NPs as such, with only a few approaches calcining the TiO_2_ NPs at temperatures above 500°C (Srinivasan et al., 2022). These bacterial-mediated synthesized TiO_2_ NPs were mainly applied in the field of biomedicine as an antimicrobial and anticancer agent, while they were also used in electronics, especially in solar cells. One major limitation of all these studies is that only two attempts were made for the photocatalytic degradation of various dyes from wastewater (Priyaragini et al., 2014; Khan and Fulekar, 2016). Other demerits in these investigations were that only a countable investigation reported the purity of the synthesized TiO_2_ NPs by any of the elemental analysis methods. One more limitation observed in all such investigations was the synthesis of TiO_2_ NPs without any dopants. Because TiO_2_ is a semiconductor material, its photocatalytic degradation property can be enhanced by adding trace elements such as Ag, Au, Pt, Sb, and tungsten (Liang et al., 2021; Pang et al., 2023). Only one investigation was carried out by Khan and Fulekar (2016), where *B. subtilis-*mediated synthesized TiO_2_ NPs were doped by using Ag, Au, and Pt (Ahmed et al., 2020; Farag et al., 2021).

In this study, the investigators have focused on the current trends in the microbial synthesis of TiO_2_ NPs. Moreover, the authors further emphasized the process and in-depth mechanism involved in the biotransformation of titanium precursors into TiO_2_ NPs in bacteria and yeast. Finally, the authors have emphasized the current and emerging applications of TiO_2_ in the biomedical field as an antimicrobial agent and for wastewater treatment. Moreover, the authors have also provided a comparative study of the synthesis and application of TiO_2_ NPs by microorganisms.

## 2. Properties of titanium dioxide nanoparticles

TiO_2_ NPs are already well described in the various pieces of literature (Yadav et al., 2022a). When it comes to the synthesis of TiO_2_ NPs by microorganisms, the synthesized TiO_2_ NPs were expected to have some unique features in comparison with TiO_2_ NPs synthesized by chemical or physical routes (Haider et al., 2019). For instance, when TiO_2_ NPs have to be used in biomedicine, especially for anticancer activity, they must be biocompatible with the host so that they may not lead to any toxicity in the host cell, which is pretty much expected in chemically or physically synthesized TiO_2_ NPs. In the case of chemically or physically synthesized TiO_2_ NPs, they must be capped or functionalized with some organic or biomolecule to increase their biocompatible nature (Rajendran et al., 2021; Yadav et al., 2021). This step can be reduced in the microbial synthesis of TiO_2_ NPs as the microorganisms have numerous microbial proteins, enzymes, and other biomolecules that act as a capping and stabilizing agent for the synthesis of TiO_2_ NPs. Moreover, due to the capping of these natural biomolecules, the biocompatibility of the TiO_2_ NPs increases. Moreover, the various functional groups present in the biomolecules on the surface of microbially synthesized TiO_2_ NPs make them naturally surface-functionalized and target-specific in comparison with the chemical or physical routes that synthesized TiO_2_ NPs (Verleysen et al., 2022).

When these microbially synthesized TiO_2_ NPs are used as an antimicrobial agent or a nano-photocatalyst, then a major drawback is their effectiveness and efficiency (Yang Q. et al., 2023). This is so because the microbially synthesized TiO_2_ NPs are capped with various microbial proteins (already proven in the literature), which hinders the activity of the TiO_2_ NPs. During the antimicrobial activity and photocatalytic effect of TiO_2_ NPs, the active sites of TiO_2_ NPs are masked by the biomolecule, resulting in less interaction between the pathogens and TiO_2_ NPs or between the pollutants and TiO_2_ NPs. Moreover, the microbially synthesized TiO_2_ NPs are capped with biological macromolecules that are larger in size, i.e., up to several kilodaltons, which increases the overall size of the TiO_2_ NPs. Due to this increased size of the biological macromolecules, the entry of large TiO_2_ NPs into the pathogens is drastically reduced due to which it would be unable to kill the pathogens photocatalytically much more effectively, ultimately making these microbially synthesized TiO_2_ NPs less effective in comparison with TiO_2_ NPs synthesized by chemical or physical route, during their application as an antimicrobial agent and nano-photocatalyst (Noh et al., 2020; Mukametkali et al., 2023).

Out of all the three polymorphs of TiO_2_, the anatase form is mainly tetragonal in structure, while rutile appears as a primitive tetragonal lattice, and brookite has an orthorhombic shape. As far as stability is concerned, rutile is the most stable, whereas anatase and brookite are metastable, i.e., both anatase and brookite, when heated to 500–700°C, get irreversibly transformed into a rutile phase. Among all the three forms of TiO_2_, the anatase phase is more photoactive in comparison with rutile and brookite, which are less photoactive (Manzoli et al., 2022). Anatase and rutile could be synthesized easily in the laboratory, but the synthesis of brookite is very difficult due to its lower thermodynamic stability. The three polymorphs of TiO_2_ exist at different temperatures in the environment (Liao et al., 2020), and the major differences between them are shown in Table 1.

**Table 1:** Differences between different polymorphs of TiO_2_ NPs.

**Parameters**	**Anatase**	**Rutile**	**Brookite**	**References**
Structure	Tetragonal structure	Primitive tetragonal lattice	Orthorhombic	Hengerer et al., 2000; Playford, 2020
Space group	I4_1_/amd (I: body-centered)	P42/mnm (P: primitive)	Pbca	Hengerer et al., 2000
Lattice parameters	*a* = 3.784 Å and *c* = 9.514 Å	*a* = 4.593 Å and *c* = 2.958 Å.	Lattice parameters of *a* = 9.1819 Å, *b* = 5.4558 Å, and *c* = 5.1429 Å	Malevu et al., 2019; Abouhaswa, 2020
Stability	Metastable: obtaining a heat of 500–700°C transformed to a rutile phase (irreversible and stable)	**–**	Metastable: obtaining a heat of 500–700°C transformed to a rutile phase (irreversible and stable)	Malevu et al., 2019; Anitha and Khadar, 2020; Manuputty et al., 2021
Photoactivity	More photoactive	Less photoactive	Less photoactive	Mikrut et al., 2020; Peiris et al., 2021; Sudrajat et al., 2022
Bandgap (ev)	3.00–3.30	~3.0	~3.1–3.4	Žerjav et al., 2022
Phototoxicity and cytotoxicity	Higher in human keratinocytes	Less	**–**	Silva et al., 2017; Amano et al., 2022; Jalili et al., 2022; Sudrajat et al., 2022; Yang F. et al., 2022

**Figure 1:**
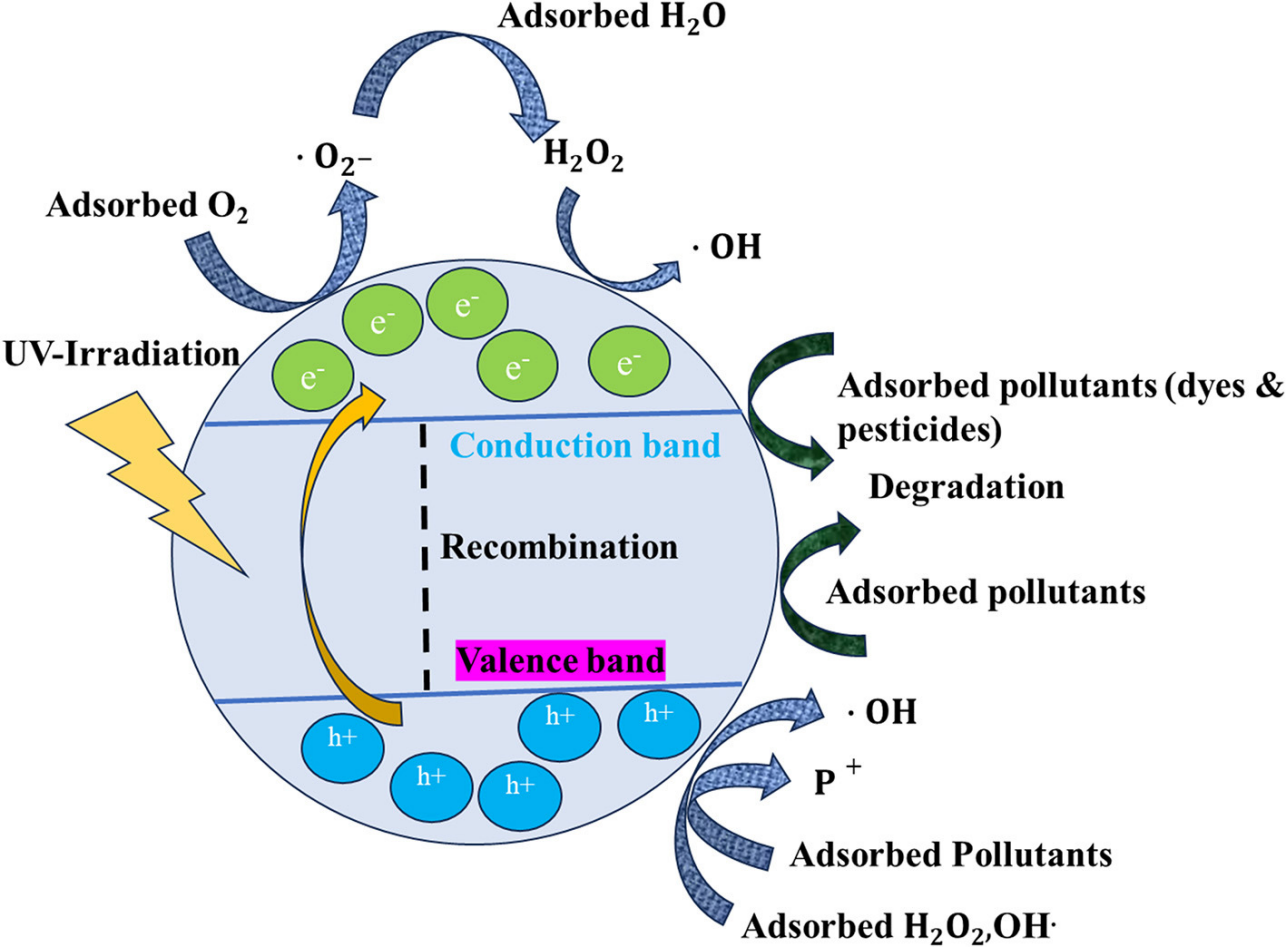
General phenomenon of photocatalysis by TiO_2_ NPs.

The photocatalytic property of TiO_2_ relies specifically on the crystal structure, morphology, and surface area. TiO_2_, being a semiconductor, has a valence band (VB) and a conduction band (CB), which play a main role in photocatalysis (Nam et al., 2019; Ullah et al., 2020; Yang H. et al., 2022; Armaković et al., 2023). TiO_2_ NPs in comparison with the bulk TiO_2_ will have a high SVR, so they will produce more reactive oxygen species (ROS) during photoexcitation (Li et al., 2014; Nunzi et al., 2015; Qutub et al., 2022). Figure 1 shows a general mechanism of photocatalysis by TiO_2_ NPs. When the TiO_2_ NPs are exposed to UV light, the electrons in the valence band get excited and reach the CB. Consequently, there is a formation of electrons (e_CB−_) and VB holes (${\text{h}}_{\text{VB}}^{+}$) (Khalafi et al., 2019), as shown in equation (2) in the TiO_2_ NPs. Furthermore, there is an interaction between these photo-excited e^−*s*^ and O_2_ dissolved in the liquid medium, which contains pollutants such as dyes and pesticides. As a consequence of this, there is the formation of superoxide radicals (•${\text{O}}_{2}^{-}$), as shown in equation (3). The pollutants present in the liquid media could be directly oxidized by the holes, as per equation (4). Furthermore, there is an interaction between the (•${\text{O}}_{2}^{-}$) and H_2_O, leading to the formation of hydrogen peroxide (H_2_O_2_) (Di Valentin, 2016; Nosaka, 2022; Samoilova and Dikanov, 2022). Furthermore, these peroxides contribute to the formation of highly reactive free hydroxyl ions (•OH). These newly formed •OH in turn interact with the pollutants, such as dyes and pesticides, available on the surface of the TiO_2_ NPs, which results in the photocatalytic degradation of these pollutants. The pollutants finally get mineralized into elements such as C, H, and O. The complete reactions and events are explained in detail by Modi et al. (2023a) and Eqs (1) to (6):


(1) $$\begin{array}{l} {\text{TiO}}_{2}+{\text{hv}}_{\left(\text{UV}-\text{ light}\right)}\to {\text{TiO}}_{2}\;\left({\text{e}}_{{\text{CB}}^{-}}\right)+{\text{h}}_{{\text{VB}}^{+}} \end{array}$$

(2) $$\begin{array}{l} {\text{e}}_{{\text{CB}}^{-}}\;+\;{\text{O}}_{2}\;\to \;{•\text{ O}}_{2^{-}} \end{array}$$

(3) $$\begin{array}{l} {\text{h}}_{{\text{VB}}^{+}}\;+{\text{OH}}^{-}\;\to \;•\text{OH} \end{array}$$

(4) $$\begin{array}{l} •\text{OH }+\text{ Dyes }\&\text{ pesticides}\to \text{Degraded products } \end{array}$$

(5) $$\begin{array}{l} {•\text{O}}_{2^{-}}\;+\text{ Dyes }\&\text{ pesticides }\to \text{ Degraded products} \end{array}$$

(6) $$\begin{array}{l} {\text{h}}_{{\text{VB}}^{+}}\;+\text{ Dyes }\&\text{ pesticides }\to \text{Degraded products } \end{array}$$

### 2.1. Mechanism of antimicrobial activity of TiO_2_ NPs

**Figure 2:**
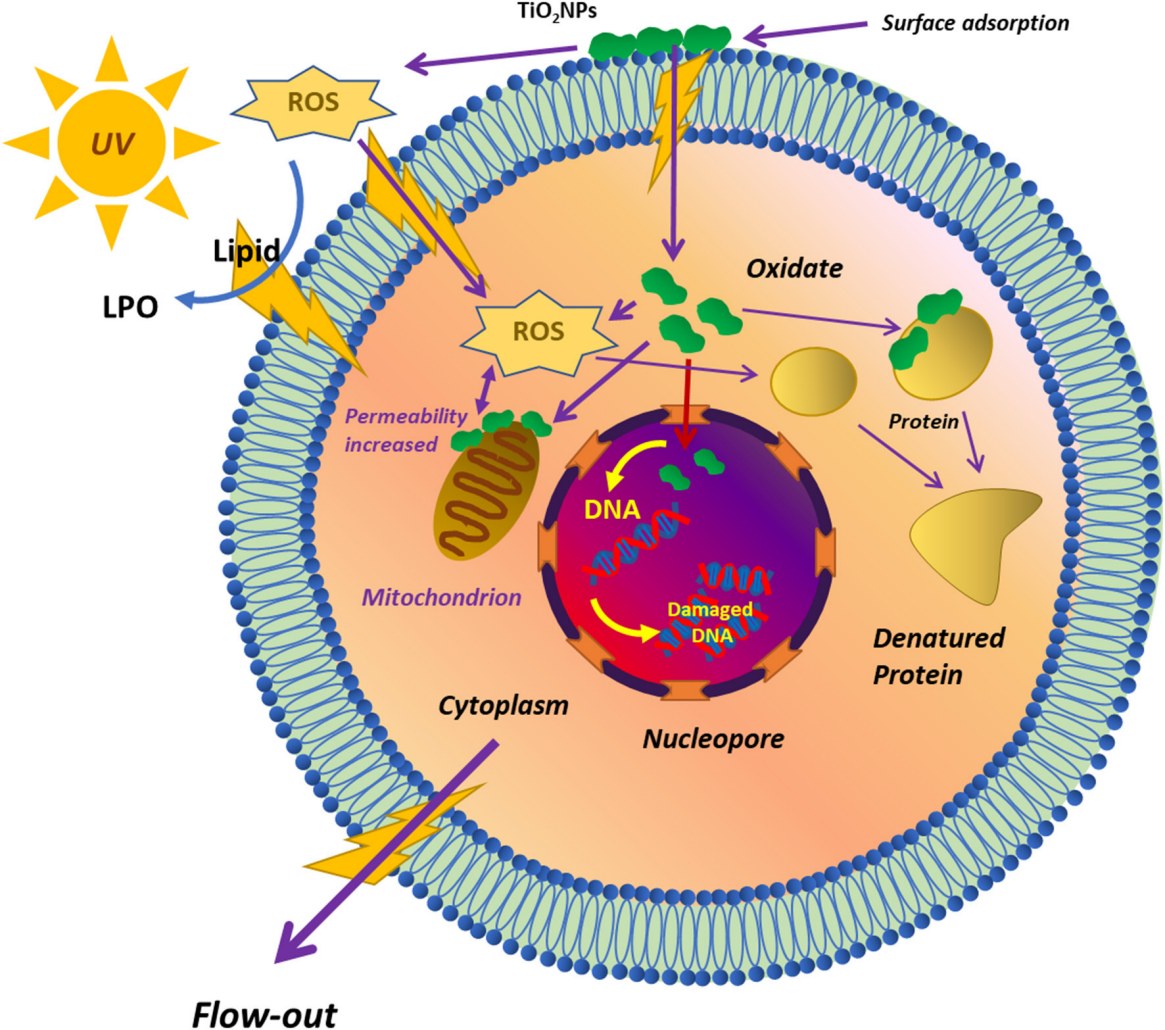
Schematic diagram of the toxicity of TiO_2_ NPs to microorganisms reprinted from Hou et al. (2019).

At the point of zero charge (pzc) at pH = 6.2, TiO_2_ NPs have negative charges on their surface, which shows a less bactericidal effect in neutral and alkaline solutions. This is so because, at these conditions, TiO_2_ NPs repel bacteria with a minus charge in the absence of light (Zhang et al., 2017; Sharma et al., 2022). During acidic pH conditions, the TiO_2_ NPs are positively charged and interact strongly with the bacterial cells, resulting in the penetration of the bacterial membrane and inducing oxidative damage accordingly (Pagnout et al., 2012). Figure 2 shows a detailed sequence involved in the toxicity of TiO_2_ NPs for microorganisms. TiO_2_ inhibits or kills microorganisms by adsorbing TiO_2_ NPs on the surface of the microorganism. There is a formation of reactive oxygen species [ROS] (${•\text{O}}_{2^{-}},•\text{OH}$) which first interacts with the lipids present on the surface of the membrane of the microorganisms (Khan et al., 2022). The interaction between lipids on the membrane and ROS leads to lipid peroxidation. In addition to this, the permeability of the lipid membrane increases. The ROS, after damaging the membrane lipids, gains entry into the cytoplasm of the microorganism, where it damages the various cellular organelles such as the mitochondria, nucleus, and their DNA and ribosomal proteins. The oxidation of proteins and DNA by ROS leads to their denaturation. Finally, all these damaged cytoplasmic contents flow out from the damaged lipid membrane, leading to the killing of microorganisms.

Earlier, several investigators have also shown the toxicity of TiO_2_ NPs on various microorganisms; for instance, Kiwi et al. exhibited that TiO_2_ NPs have a bactericidal effect on *Escherichia coli* by direct contact in dark conditions. During this process, the cell wall gets damaged because of the electrostatic attraction between the TiO_2_ NPs and the minus-charged bacterial cell wall at a pH close to but below pzc (Kiwi et al., 2014). The antibacterial activity of TiO_2_ NPs is mainly due to the production of ROS in the presence of UV light, suggesting that the bactericidal effect is due to UV light and not due to TiO_2_ NPs (Vatansever et al., 2013). TiO_2_ NPs have also shown potential for the killing of multidrug-resistant bacteria through the reactive radicals produced by electron–hole pairs upon UV irradiation (Kubacka et al., 2014). The inactivation of drug-resistant bacteria by a photocatalytic material such as TiO_2_ NPs relies on the power and irradiation time of UV-A light (Tsai et al., 2010). Hence, the disinfection method needs a high-power UV source to excite TiO_2_ NPs, and in visible light, there are fewer bactericidal uses owing to their ineffective photoexcitation. Due to this, in an indoor environment where there is a small amount of UV light, the efficiency of TiO_2_ NPs against microorganisms is limited. As a result, the development of such TiO_2_ NPs that may be activated with visible light in addition to their excellent antibacterial properties is one of the most urgent needs in the medical and industrial sectors.

## 3. Synthesis of TiO_2_ NPs

TiO_2_ NPs could be synthesized by all three possible routes: chemical, physical, and biological. The physical approaches involve thermal evaporation, pulsed discharge plasma, reactive DC magnetron sputtering, pulsed laser deposition (PLD), and the chemical gas-phase atomic layer deposition (ALD) method. Recently, Wahyudiono et al. (2022) synthesized TiO_2_ NPs by using high-voltage discharge plasma under pressurized argon environmental conditions. Kumi-Barimah et al. synthesized a thin film of TiO_2_ by PLD at a substrate temperature of 25, 400, and 600°C. In this study, the investigators obtained a size of ~35 nm nanoparticulates (Kumi-Barimah et al., 2020). Dreesen et al. (2009) synthesized 19 nm-sized TiO_2_ NPs by using reactive DC magnetron sputtering.

The thin film of TiO_2_ developed by the physical approach is suitable for dye-sensitized solar cells, microelectromechanical systems, and electroluminescent gadgets (Orlianges et al., 2012; Bai et al., 2014). The chemical approaches involve coating (dip, spin, and spray), co-precipitation, ultrasonication wet impregnation, photoreduction, hydrothermal and solvothermal processing, electrochemical anodization and electrospinning, and sol-gel (Orlianges et al., 2012; Zhu et al., 2017; Johari et al., 2019). Latha and Lalithamba (2018) synthesized spherical-shaped anatase-phased TiO_2_ NPs by using a hydrothermal method, which was calcinated at 400°C. Vajedi and Dehghani (2016) synthesized ~12 nm TiO_2_ NPs by using a solvothermal method, which was capped by using diethyl oxalate (Vajedi and Dehghani, 2016). Buraso et al. (2018) synthesized TiO_2_ NPs of size 11.3 to 27.4 nm under varying calcination temperatures of 400–700°C. The precursor used here was titanium (IV) isopropoxide, and the synthesis method was a simple precipitation method. Oh et al. (2005) synthesized spherical-shaped TiO_2_ NPs of size 14–22 nm by applying ultrasonication. Jongprateep et al. synthesized 48–85 nm-sized TiO_2_ NPs by using the sol-gel method. In this study, the precursor used for the synthesis of TiO_2_ NPs was titanium (IV) isopropoxide (TTIP) (Jongprateep et al., 2015).

The TiO_2_ NPs/thin films developed by chemical approaches are mainly suitable for antimicrobial activity. Moreover, such methods are easy and convenient. In addition to this, several investigators have reported that by using such chemical routes, it is possible to synthesize larger quantities of TiO_2_ NPs than by using physical methods in comparison with the physical processing route.

### 3.1. Microbial synthesis of titanium dioxide NPs

Microbes have various enzymes, metabolites, and pigments that are responsible for transforming metal ions into metal oxides or metallic NPs (Choudhary et al., 2023). To date, metallic NPs have been synthesized by bacteria, fungi, actinomycetes, algae, and viruses (Choudhary et al., 2023; Dadhwal et al., 2023). Several investigators have synthesized TiO_2_ NPs by using all these types of microorganisms. The TiO_2_ NPs synthesized from the microbes have several advantages and features, such as biocompatibility, being eco-friendly, and being non-toxic (Ahmad and Kalra, 2020; Yadav et al., 2020b; Tripathy et al., 2023). The formation of TiO_2_ NPs by microorganisms is explained below in detail.

### 3.2. Bacterial synthesis of titanium dioxide NPs

To date, bacteria have been used most extensively for the synthesis of TiO_2_ NPs. Bacteria are rich in several biomolecules that transform the Ti salts into TiO_2_ NPs (Yadav et al., 2020b). There are several bacteria that could synthesize TiO_2_ NPs, either intracellularly or depending on the nature of the bacteria (Farag et al., 2021). Due to changes in the environment, the bacteria become resistant to some of the metals that help them in the synthesis of NPs. The natural defense system is present in bacteria, which makes them resistant to harsh conditions. Bacterial-mediated synthesis can take place by all three means, i.e., by whole cells, supernatants, and extracts. Supernatants can be taken after the centrifugation of the bacterial culture, which has enzymes, microbial proteins, and metabolites secreted by the bacteria or released after centrifugation. Moreover, TiO_2_ NPs can also be synthesized by using bacterial pellets after dispersing them with distilled water and providing a titanium precursor (Liou and Chang, 2012). The transformation of Ti^3+^ ions into TiO_2_ NPs by microorganisms involves three basic steps, i.e., trapping, bioreduction, and capping. First, the Ti^3+^ ions get trapped by the bacteria in the aqueous solution or surrounding medium. Furthermore, with the help of enzymes and proteins, the trapped Ti^3+^ ions get reduced into TiO_2_ NPs. From the investigations, it has been proven that the microbial proteins having functional groups –NH_2_, –SH, –COOH, and –OH help in stabilizing the synthesized TiO_2_ NPs (Wang B. et al., 2021; Hazem Najem et al., 2023). These functional groups generally provide a site for the binding of metallic ions such as Ti^3+^ ions in addition to a capping agent or stabilizing agent. Immediate to this, there is the reduction of Ti^3+^ ions into its NPs. The reduction of Ti^3+^ ions takes place either on the cell wall or in the periplasmic space. During this step, electrons move from reduced compounds to inorganic compounds. This promotes the bioreduction process in bacteria for NPs. Finally, the reduced TiO_2_ NPs get capped by the various biomolecules present in the bacteria, acting as a natural capping agent for the synthesized TiO_2_ NPs (Baig et al., 2020). Capping helps in maintaining the stability of NPs, which is an important factor. There are a few examples where these microbial proteins acted as the major reducing or capping agents at the time of formation and stabilization of TiO_2_ NPs (Lahiri et al., 2021). Jha et al. (2009) and Jayaseelan et al. (2013) described a similar mechanism in *Lactobacillus* and *Aeromonas hydrophila*, respectively.

Jha et al. (2009) hypothesized a series of chemical reactions involved in the formation of TiO_2_ NPs by using *Lactobacillus* bacteria.


(7) $$\begin{array}{l} {\text{C}}_{6}{\text{H}}_{12}{\text{O}}_{6}\to {\text{CH}}_{3}-\text{CO}-\text{COOH}↔{\text{CH}}_{3}.\text{CH}\left(\text{OH}\right).\text{COOH} \end{array}$$

(8) $$\begin{array}{l} {\text{NaHCO}}_{3}↔{\text{Na}}^{+}+{\text{HCO}}_{3}^{-} \end{array}$$

(9) $$\begin{array}{l} {\text{HCO}}_{3}^{-}↔{\text{OH}}^{-}+{\text{CO}}_{2}\; \end{array}$$

(10) $$\begin{array}{l} \text{TiO}.{\left(\text{OH}\right)}_{2}\to {\text{TiO}}_{2}↓\;+\;{\text{H}}_{2}\text{O } \end{array}$$

**Figure 3:**
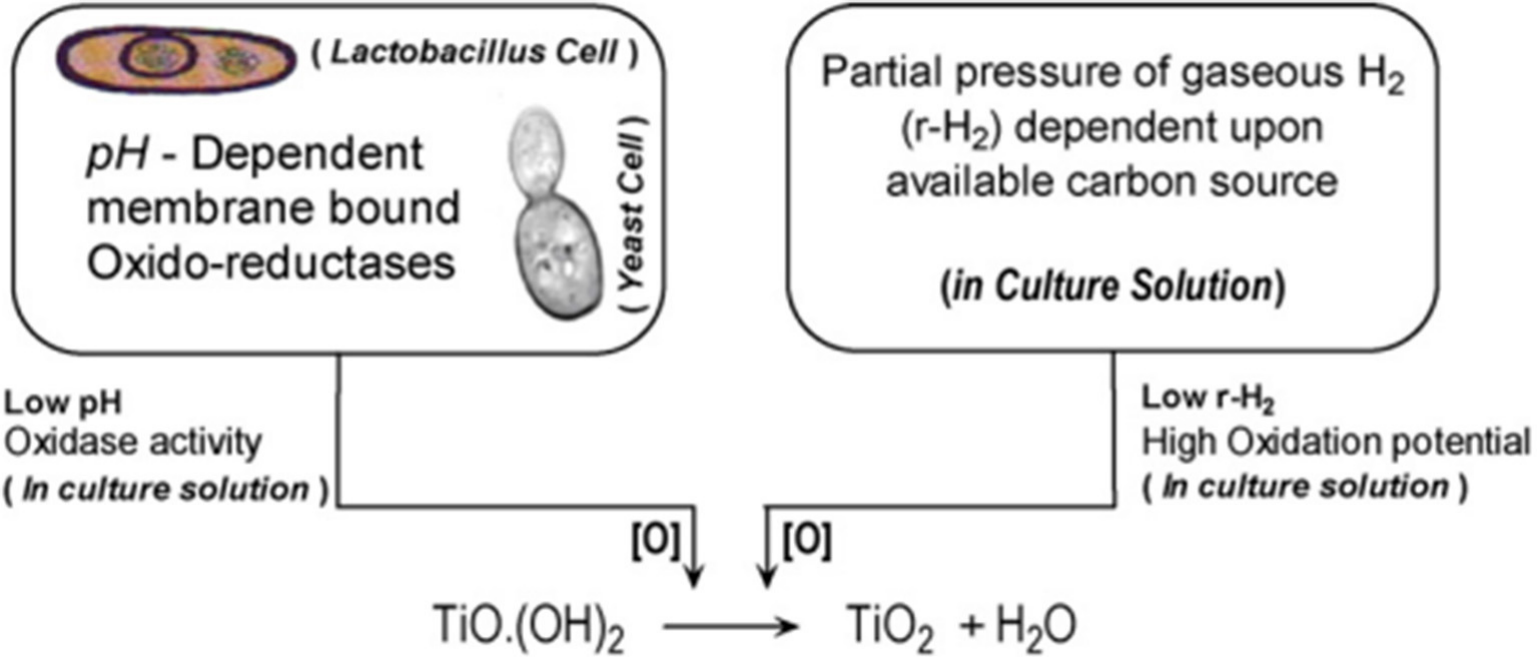
Schematics for the biosynthesis of n-TiO_2_ adapted with permission from Jha et al. (2009).

Moreover, Jha et al. also deduced a schematic for the biosynthesis of TiO_2_ NPs, which is shown below in Figure 3. *Lactobacillus* is said to have pH-dependent membrane-bound oxidoreductases, which exhibit oxidase activity at lower pH. Due to this, the titanium hydroxide gets converted into TiO_2_ NPs by producing H_2_O as a by-product (Jha et al., 2009).

**Figure 4:**
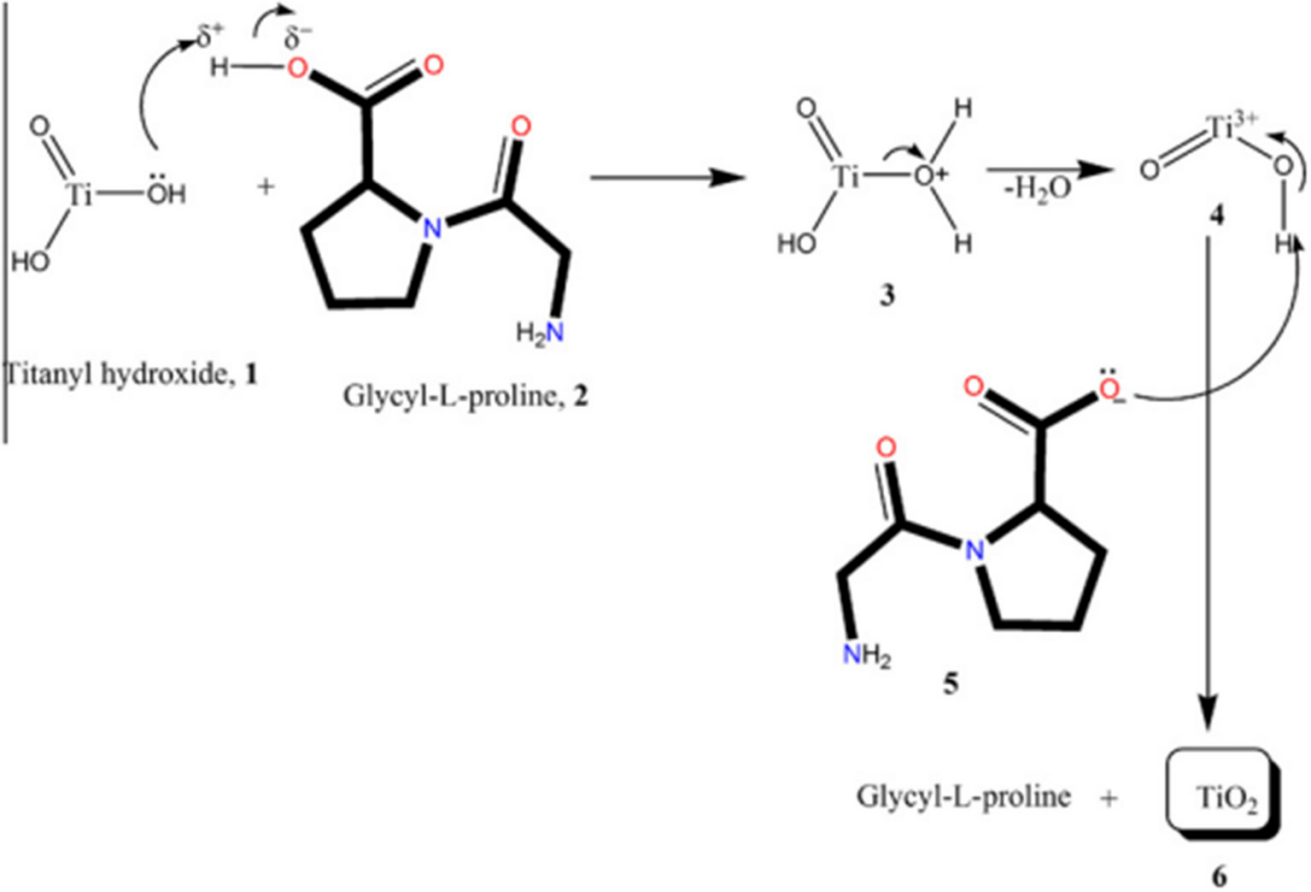
Possible mechanisms for the synthesis of nanosize TiO_2_ particles adapted from Jayaseelan et al. (2013).

Jayaseelen et al. suggested another possible mechanism for the synthesis of TiO_2_ NPs by using *A. hydrophila*, which is shown in Figure 4. According to the investigators, the secondary metabolites produced by *A. hydrophila*, especially glycyl-L-proline and compounds having –COOH and –C=O as a functional group, have a major function in the synthesis of TiO_2_ NPs. Furthermore, investigators suggested that the Ti precursor (titanyl hydroxide) can be dehydrated by the glycyl-L-proline to give TiO_2_ NPs once the broth of *A. hydrophila* interacts with the precursor at ~30°C for 24 h. Furthermore, the investigators suggested that the synthesis of TiO_2_ NPs by *A. hydrophila* could be accomplished in a series of steps. In the first step, one of the lone pairs of electrons present in O_2_ picks up an H^+^ ion from the glycyl-L-proline. In the second step, there is protonation of TiO(OH)_2_, while in the third step, the protonated TiO(OH)_2_ loses an H_2_O molecule, resulting in the formation of Ti^3+^ ions. Finally, an intermediate compound (5) pulls off a H^+^ ion from the Ti^3+^. In this study, the investigator concluded that the stability of the synthesized TiO_2_ NPs is mainly due to the -COOH group containing water-soluble compounds. All these hypotheses and suggestions were based on the gas chromatography-mass spectroscopy (GCMS) analysis of the *A. hydrophila* broth culture. The GCMS showed four major compounds, namely uric acid (2.95%), glycyl-L-glutamic acid (6.90%), glycyl-L-proline (74.41%), and l-Leucyl-d-leucine (15.74%). Moreover, investigators concluded the presence of mainly two functional groups in the sample, namely –COOH and –C=O. So, the investigators finally concluded that these two compounds may play a significant role in the synthesis of TiO_2_ NPs as stabilizing and capping agents (Jayaseelan et al., 2013).

From the various pieces of literature, it has been found that to date, 9–10 different bacteria have been used for the synthesis of TiO_2_ NPs, for instance, three species of *Bacillus* (*B. subtilis, Bacillus mycoides*, and *Bacillus amyloliquefaciens*), two *Lactobacillus* (*Lactobacillus johnsonii* and *Lactobacillus* sps), one *Propionibacterium jensenii, S. aureus, Planomicrobium* sps*, Halomonas elongata* IBRC-M 10214, and *A. hydrophila*. Table 2 shows the various previous attempts by investigators to synthesize TiO_2_ NPs. To date, various investigators have synthesized TiO_2_ NPs by using bacteria and fungi; for instance, Jayaseelan et al. (2013) synthesized 28–84 nm-sized TiO_2_ NPs using *A. hydrophila*. Landage et al. synthesized spherical-shaped 20 nm TiO_2_ NPs by using *S. aureus* and later applied them for antibacterial activity. Al-Zahrani et al. (2018) used *L. johnsonii* for the synthesis of 4–9 nm-sized TiO_2_ NPs. Among bacteria, *Bacillus* species have been used earlier for the synthesis of TiO_2_ NPs (Khan and Fulekar, 2016). Taran et al. (2018) synthesized 104.63 ± 27.75 nm TiO_2_ NPs by using *H. elongata* IBRC-M 10214. As far as Bacillus species are concerned, it was earlier used by Khan and Fulekar (2016) and Kirthi et al. for the synthesis of TiO_2_ NPs. Kirthi et al. synthesized oval to spherical shapes of size 67–77 nm by using *B. subtilis*. *Bacillus mycoides* has been recently utilized for the synthesis of TiO_2_ NPs at low temperatures according to Yamauchi et al. (2011) and Órdenes-Aenishanslins et al. (2014). Khan and Fulekar (2016) synthesized spherical TiO_2_ NPs by using *B. amyloliquefaciens*, where the size of the TiO_2_ NPs varied from 22.11 to 97.28 nm. The authors further revealed that alpha-amylase is accountable for the synthesis of TiO_2_ NPs (Khan and Fulekar, 2016).

**Table 2:** Bacterial-mediated synthesis of TiO_2_ NPs by using different titanium precursors and conditions.

**Titanium precursors and their strength**	**Bacteria used**	**Gram +ve/–ve**	**Shape of TiO_2_ NPs**	**Size (nm)**	**Elements**	**Technique applied**	**Temperature (°C)**	**References**
TiO(OH)_2_	*Halomonas elongata* IBRC-M 10214	Rod-shaped, +ve	Spherical	104.63 ± 27.75		Steam bath heating of 24-h-old culture, time: 10–20 min, followed by incubation	60	Taran et al., 2018
TiO(OH)_2_, 0.025 M	Lactobacillus	Rod, +ve	Spherical	24.63 ± 0.32		Steam bath heating of 24-h-old culture, time: 10–20 min, followed by incubation	60	Jha et al., 2009
TiO(OH)_2_ 0.025 M	*Bacillus subtilis*	+ ve	Spherical, oval	66–77		Steam bath heating of 24-h-old culture at 60°C 10–20 min, followed by incubation	60	Vishnu Kirthi et al., 2011
TiO(OH)_2_, 5 mM	*Aeromonas hydrophila*	–ve	Spherical and uneven	28–54		Shaking in an incubator at 120 rpm at 30°C for 24 h	30	Jayaseelan et al., 2013
TiO(OH)_2_, 0.025 M	*Propionibacterium jensenii*	+ve	Smooth, spherical	15–80	Ti: 54.73 and O: 45.27	Steam bath heating of 24-h-old culture at 60°C ~20 min, followed by incubation	60	Babitha and Korrapati, 2013
TiO(OH)_2_, 0.0025 M	*Staphylococcus aureus*	+ve	Spherical and oval	20		Steam bath heating of 24-h-old culture at 60°C 10–20 min, followed by incubation	60	Landage et al., 2020
0.5 g of Potassium hexafluorotitanate in 500 ml in ddw	*Bacillus subtilis* (*FJ460362*)	+ve	Spherical	10–30		Sonication, incubation with shaking, centrifugation, and calcination	37	Dhandapani et al., 2012
0.025 g of TiO_2_	*Planomicrobium* sp.	+ve	Agglomerated, irregular shape	More than 100		Steam bath heating of 24-h-old culture at 60°C 10–20 min, followed by incubation	60	Chelladurai et al., 2013
0.025 M TiSO_4_	*B. amyloliquifaciens*	+ve	22.11–97.28 (by TEM)	Spherical	Ti: 48.75 and O: 43.15	Incubation, for 1 day at 37°C, centrifugation, calcination at 500°C for 3 h	37	Khan and Fulekar, 2016
TiO_2_ (0.025m)	*Lactobacillus johnsonii*	+ve	Irregular, agglomerated	4–9		Incubation, supernatant, shaker at 37°C, centrifugation and drying at 50°C for 1 h	37	Al-Zahrani et al., 2018
TiO(OH)_2_	*Paenibacillus* sp. HD1PAH	+ve	Spherical	Average size by DLS 17.11 nm		Grown in nutrient broth. Steam bath heating of 24-h-old culture at 60°C 10–20 min, followed by incubation		Chakravarty et al., 2023
Titanyl hydroxide	*Bacillus mycoides*	+ve	Spherical	40–60		Incubation at 37°C followed by lowering of temp: (20–25°C)		Órdenes-Aenishanslins et al., 2014

From the bacterial synthesis of TiO_2_ NPs, it has been concluded that the majority of Gram-positive bacteria have been used for the synthesis of TiO_2_ NPs. Among all the approaches, Ti(OH)_2_ has been used maximally by investigators for the synthesis of TiO_2_ NPs.

### 3.3. Synthesis of TiO_2_ NPs by actinomycetes

Actinomycetes are higher-GC-containing fungi that are mainly used for the production of antibiotics. In addition to this, several investigators synthesized TiO_2_ NPs by using actinomycetes. Some of the most recent examples are highlighted below. Agceli et al. synthesized spherical-shaped, 30 to 70 nm TiO_2_ NPs by utilizing *Streptomyces* sp. HCl. The developed TiO_2_ NPs were evaluated for their antimicrobial activity against pathogenic bacteria *S. aureus* ATCC 29213*, E. coli* ATCC 35218*, Candida albicans* ATCC 10231, and fungi *A. niger* ATCC 6275. Investigators concluded that the TiO_2_ NPs showed higher antimicrobial properties against bacteria than fungi (Agçeli et al., 2020).

An investigation led by Priyaragini synthesized TiO_2_ NPs from the precursor's titanium hydroxide by using marine actinobacteria, i.e., *Streptomyces bluensis*. This particular strain was collected from the coastal area of Tamil Nadu, India. The spherical-shaped TiO_2_ NP average size was 37.54 nm, which was further used for the photocatalytic degradation of Acid Red 79 (AR-79) and Acid Red 80 (AR-80) azo dyes with an efficiency of 84 and 85%, respectively (Priyaragini et al., 2014).

### 3.4. Fungal-mediated synthesis of TiO_2_ nanoparticles

In comparison with bacteria, fungi are most preferred by scientists for the biosynthesis of TiO_2_ NPs, as most of the fungi are extracellular, allowing easy recovery of the NPs. In addition to this, large-scale production and economic feasibility are the other factors for the fungi-mediated synthesis of TiO_2_ NPs (Irshad et al., 2021). Various enzymes present in the fungus make them adaptable to different environmental conditions. Enzymes are accountable for the reduction of Ti^3+^ ions into oxide, and NADPH acts as a co-factor in this mechanism. There are various species for the synthesis of NPs, such as *A. niger, Aspergillus flavus*, and *Fusarium oxysporum*. Jha and their team synthesized 12.57 ± 0.22 nm, spherical-shaped TiO_2_ NPs by using *Saccharomyces cerevisiae* (yeast) by using TiO(OH)_2_, 0.025 M as a precursor. In this study, the investigators first heated the 24-h-old yeast culture on a water bath heater at 60°C for 10–20 min, which was further incubated at 28–30°C for the formation of TiO_2_ NPs. Furthermore, the culture was harvested after 48–72 h by centrifugation. The characterization of the yeast-mediated synthesized TiO_2_ NPs by Fourier transform infrared (FTIR) bands exhibited typical bands in the region of 400–3,600 cm^−1^. In addition to this, X-ray diffraction (XRD) analysis revealed the two major intensity peaks at two thetas of 25 and 28°, which were assigned to anatase (101) and rutile, respectively. In this study, the investigators obtained an average particle size of ~18 nm by XRD, which was in close agreement with the result obtained by TEM. Investigators parallelly used *Lactobacillus* for the TiO_2_ NPs and concluded that under similar conditions, yeast produced smaller-sized TiO_2_ NPs, i.e., 18 nm, in comparison with *Lactobacillus* (30 nm). This is so because yeast is eukaryotic in nature and has a better level of organization at the cellular level. Furthermore, the investigator suggested a series of chemical reactions involved in the biotransformation of titanyl hydroxide to TiO_2_ NPs by yeast. During the TiO_2_ NPs synthesis by yeast, the glucose sugar molecules are converted to ethanol, acetaldehyde, and finally to acetic acid. Furthermore, this acetic acid gets ionized into acetate ions and H^+^ ions. Furthermore, sodium hydrogen carbonate present in the medium on ionization produces Na^+^ ions and carbonate ions. Furthermore, these hydrogen ${\text{HCO}}_{3}^{-}$ ions split to produce hydroxyl ions and carbon dioxide gas. Finally, the OH ions generated in the previous reactions react with Ti^3+^ ions to form TiO_2_ NPs by releasing a water molecule.


(11) $$\begin{array}{l} {\text{C}}_{6}{\text{H}}_{12}{\text{O}}_{6}\to {\text{C}}_{2}{\text{H}}_{5}\text{OH}\to {\text{CH}}_{3}\text{CHO}\to {\text{CH}}_{3}\text{COOH} \end{array}$$

(12) $$\begin{array}{l} {\text{CH}}_{3}\text{COOH}↔{\text{CH}}_{3}{\text{COO}}^{-}+{\text{H}}^{+} \end{array}$$

(13) $$\begin{array}{l} {\text{NaHCO}}_{3}↔{\text{Na}}^{+}+{\text{HCO}}_{3}^{-} \end{array}$$

(14) $$\begin{array}{l} {\text{HCO}}_{3}^{-}↔{\text{OH}}^{-}+{\text{CO}}_{2} \end{array}$$

(15) $$\begin{array}{l} \text{TiO}.{\left(\text{OH}\right)}_{2}\to {\text{TiO}}_{2}↓\;+\;{\text{H}}_{2}\text{O}.\; \end{array}$$

The investigators also explained the mechanism of the formation of TiO_2_ NPs by yeast, which was almost similar to the mechanism reported in the case of *Lactobacillus*. As per the investigations, it was found that yeast has oxidoreductase and quinones, which are present on the membrane surface and in the cytosol too. Being pH-sensitive, oxidoreductase gets activated at lower pHs, while at higher pH values, it activates the reductase. While another molecule, quinone, facilitates the redox reaction due to tautomerization. When TiO(OH)_2_ is added to an aqueous medium containing yeast, tautomerization of quinones and oxidases (sensitive at low pH) occurs, making molecular O_2_ available for biotransformation. Once TiO(OH)_2_ enters the cytosol, it will trigger the family of oxygenases present in the endoplasmic reticulum (ER). ER is known for detoxification at the cellular level by the phenomenon of oxidation/oxygenation (Jha et al., 2009).

Bansal et al. reported the biosynthesis of titania from *A. niger* by using K_2_TiF_6_ as a precursor. In this study, first, the fungus was grown in malt, glucose, yeast, and peptone (MGYP) medium, and later on, mycelia were separated from the medium, washed with deionized water, followed by an addition of 20 g of wet mycelia of fungi and 100 ml of aqueous solutions of K_2_TiF_6_, and kept under shaking conditions for 1 day. The investigators further characterized the sample by transmission electron microscopy (TEM) and scattering area electron diffraction (SAED) and found that the synthesized titania was spherical-shaped with sizes varying from 6 to 13 nm with an average size of 10.2 ± 0.1 nm. The SAED pattern showed a sharp ring for the calcinated TiO_2_ NPs. This indicated the formation of brookite structures for titania. From the XRD and TEM, it was found that the d-values obtained (3.47 A°, 2.24 A°, 1.97 A°, and 1.28 A°) match reasonably well with the standard *d*-values (3.46 A°, 2.24 A°, 1.97 A°, and 1.28 A°) for the 111, 022, 032, and 004 planes, respectively, of the brookite polymorph of TiO_2_ (Bansal et al., 2005).

Tarafdar et al. (2013) synthesized TiO_2_ NPs of size <100 nm by using *Aspergillus tubingensis*. *Aspergillus niger*-mediated synthesis of TiO_2_ NPs of size 73.58–106.9 nm was reported by Durairaj et al. (2014). Rajakumar et al. synthesized oval-shaped TiO_2_ NPs of size 62–74 nm from *A. flavus* and assessed their antimicrobial activity against *E. coli*. The FTIR investigations by Rajakumar et al. (2012) observed a band at 590 cm^−1^, which is attributed to the Ti-O bonds.

**Figure 5:**
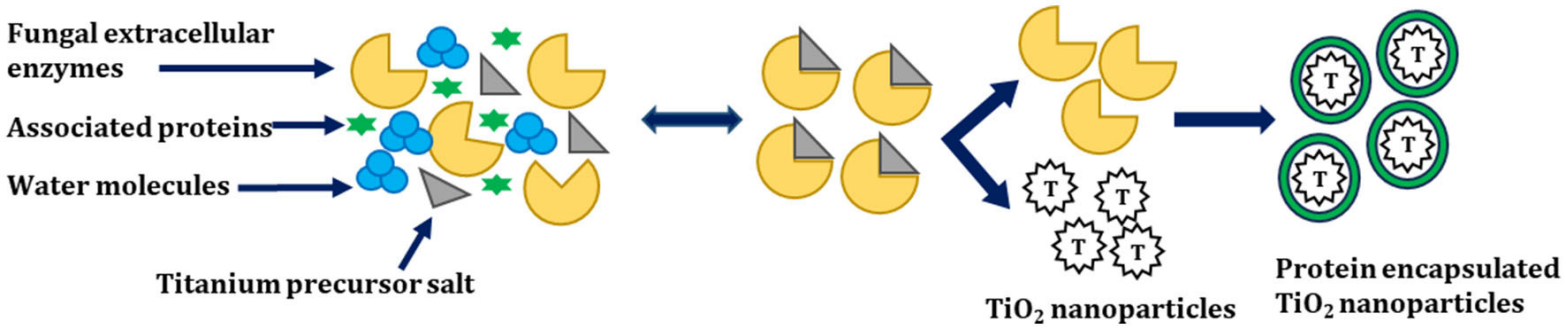
Mechanism for the biosynthesis of TiO_2_ NPs adapted from Raliya et al. (2015).

Raliya et al. synthesized spherical TiO_2_ NPs whose size varied from 12 to 15 nm by using *A. flavus* TFR 7 with a purity of ~94% (atomic wt.%). Furthermore, the investigators assessed its physiological activity on mung bean (*Vigna radiata* L.). Investigators found that the plants treated with TiO_2_ NPs in comparison with micron-sized TiO_2_ were comparatively taller. In this study, the authors proposed a mechanism for the fungal-mediated synthesis of TiO_2_ NPs, which is shown below (Figure 5).

In this study, *A. flavus* TFR 7 secretes enzymes outside and synthesizes the TiO_2_ NPs extracellularly. The synthesized TiO_2_ NPs obtained over here were highly pure, monodisperse nanoparticles, and free from cellular debris. Moreover, the downstream processing of TiO_2_ NPs from *A. flavus* TFR 7 was very easy. Furthermore, investigators suggested a mechanism for the biotransformation of TiO_2_ NPs from titanium precursors. The first step involves the secretion of extracellular enzymes by the fungi, which encapsulate the TiO_2_ NPs by capping protein, which increases the stability of TiO_2_ NPs. In addition to this, the associated proteins may help in the biotransformation of precursor salt (Raliya et al., 2015).

Chinnaperumal et al. (2018) extracellularly synthesized regular spherical-shaped 60–86.67 nm TiO_2_ NPs by using *Trichoderma viride* and assessed their larvicidal, antifeedant, and pupicidal activity against *Helicoverpa armigera*. For the synthesis, the investigators used 10, 50, and 100% of fungal supernatant and mixed it with 20 ml of 5 mM TiO(OH)_2_, followed by incubation along with shaking at 120 rpm for 24 h at 30°C. From the XRD investigation, peaks were obtained at peaks at 27.41°, 32.34°, 44.27°, 54.29°, and 64.55° which corresponded to (1 1 0), (1 0 0), (1 1 1), (2 1 1), and (3 0 1), indicating rutile form. From the FTIR analysis, investigators found bands at 3,430.48 cm^−1^ (O–H stretch), 2,923.87 cm^−1^ (O–H stretch; carboxylic acids), 2,148.48 cm^−1^ (–C=C– stretch; alkynes), 1,729.88 cm^−1^ (C=O stretch; carboxylic acids), 1,648.25 cm^−1^ (–C=C– stretch; alkenes), 1,424.81 cm^−1^ (C–C stretch), 1,375.87 cm^−1^ (C–H rock; alkanes), 1,317.13 cm^−1^ (C–N stretch; aromatic amines), 1,252.45 cm^−1^ (C–N stretch), 1,038.87 cm^−1^ (C–O stretch; alcohols, carboxylic acids, esters, and ethers), and 563.83 cm^−1^ (C–Cl stretch; alkyl halides) (Chinnaperumal et al., 2018).

Heitzschold et al. stated that the synthesis of NPs takes place through the action of the NADPH factor, but studies have found that changes in pH, temperature, incubation time, type of fungi, and the source used can definitely affect the morphology of NPs. Rehman et al. reported the synthesis of TiO_2_ and Ag NPs by using the wild mushroom *Fomitopsis pinicola*. Furthermore, the investigator evaluated the potential of both synthesized NPs against *E. coli* and *S. aureus*. Moreover, NPs were also evaluated on the human colon cancer cell line (HCT) by minimum inhibitory concentration/minimum bactericidal concentration (MIC/MBC) and MTT assays (Rehman et al., 2020).

Sathiyaseelen et al. synthesized TiO_2_ NPs by using the endophytic fungus *Paraconiothyrium Brasiliense*, which was further assessed for its antibacterial activities. The synthesized TiO_2_ NPs were spherical in shape, whose size was ~57.39 ± 13.65 nm by TEM, whereas the average hydrodynamic size was (68.43 ± 1.49 d. nm), and the zeta potential was found to be (−19.6 ± 1.49 mV) (Sathiyaseelan et al., 2022).

In one of the most recent attempts, Survase and Kanase (2023) synthesized TiO_2_ nanospheres from precursor titanium chloride (TiCl_3_) by using *Aspergillus eucalypticola* SLF1. Furthermore, the investigators used TiO_2_ for the antimicrobial activity and dye removal (reactive blue 194) (Survase and Kanase, 2023).

### 3.5. Synthesis of TiO_2_ NPs by algae

**Figure 6:**
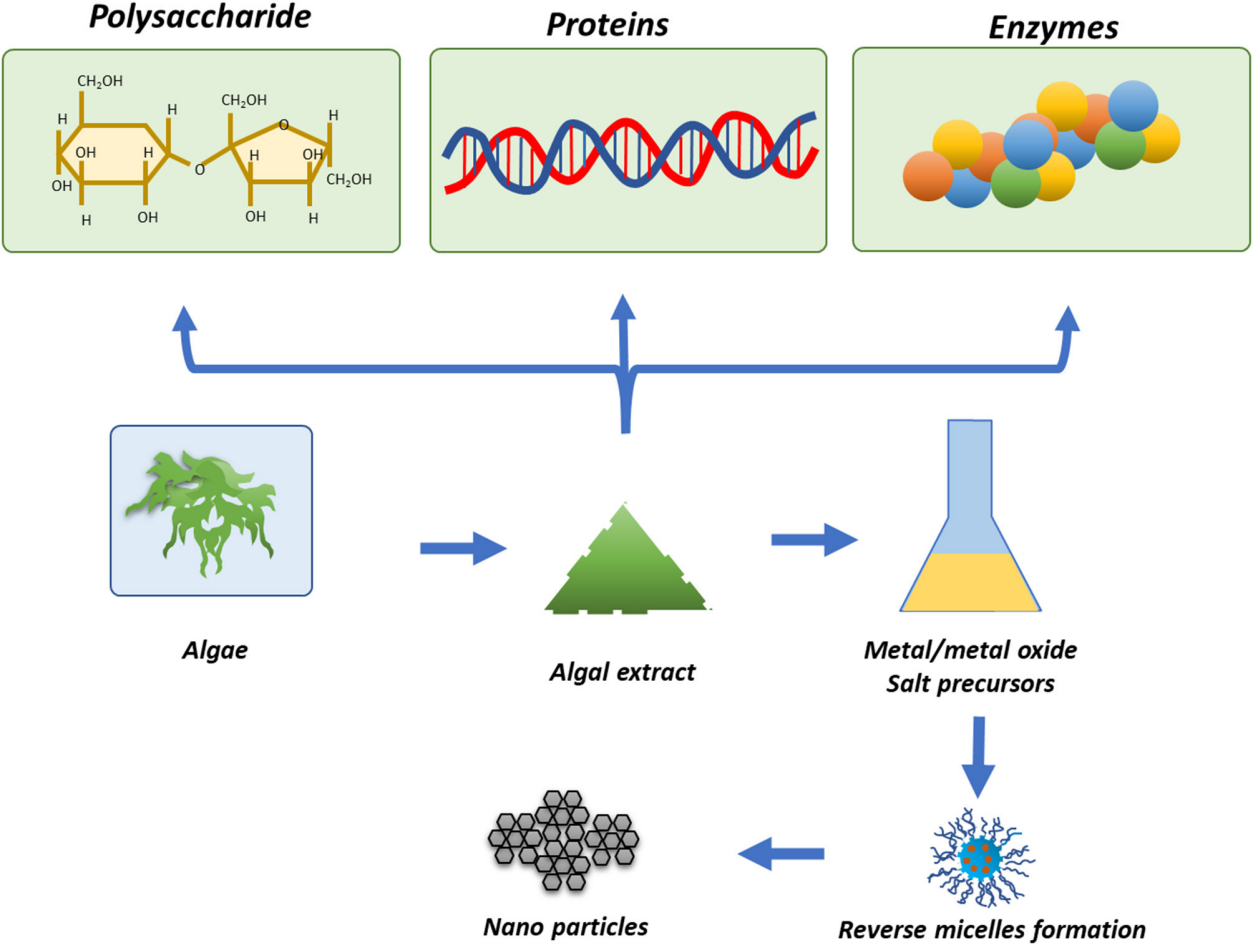
Algae-mediated synthesis of metal/metal oxide NPs reproduced from Narayanan and Sakthivel (2011).

One of the most important groups of photosynthetic organisms is algae. Various pieces of literature have shown that algae have a tendency to accumulate higher levels of heavy metals, so this property can be exploited for the biosynthesis of metallic and metal oxide NPs (Priyadarshini et al., 2019; You et al., 2021). Algae have been widely used for the synthesis of TiO_2_ NPs due to their easy access and efficacy. In addition to enzymes and proteins, algae also have carotenoids and various photosynthetic pigments, which play an important role in the phyco-assisted synthesis of TiO_2_ NPs. However, the algal-mediated synthesis of TiO_2_ NPs is not as developed as for bacteria. Moreover, NPs could also be synthesized by algae by using their extracts/supernatant, which contain secondary metabolites. Hifney et al. (2022) synthesized TiO_2_ NPs by using the algae *Spirulina platensis*. Vasanth et al. (2022) synthesized spherical-shaped TiO_2_ NPs of 90 to 150 nm size by using *S. platensis* extract. Figure 6 shows the various approaches for the physio-assisted synthesis of TiO_2_ NPs.

In one of the most recent investigations, Mathivanan et al. (2023) synthesized TiO_2_ NPs from *Sargassum wightii* (seaweed) and evaluated their potential for killing the larvae of vectors responsible for causing malaria and filariasis. In another attempt, Balaraman et al. (2022) synthesized negatively charged cubic, square, and spherical-shaped, ~50–90 nm-sized TiO_2_ NPs by using *Sargassum myriocystum*. Furthermore, the investigator assessed the antimicrobial activity of the synthesized TiO_2_ NPs.

### 3.6. Synthesis of TiO_2_ NPs by virus

Viruses are a significant example of the synthesis of nanosized particles. To date, viruses have been used for the synthesis of nanotubes, nanorods, etc. (Koudelka et al., 2015). It has been shown by studies and experiments that plant viruses and some bacteriophages are easy to isolate and process further. All the parts of the virus cannot be used to formulate nanoparticles, and the reason still needs to be studied perfectly. Zhou et al. (2014) reported the formation of chalcogenide nanocrystals inside the genetically modified virus-like particles. Órdenes-Aenishanslins et al. also emphasized the importance of viruses in the synthesis of various types of NPs (Órdenes-Aenishanslins et al., 2014).

## 4. Characterization of TiO_2_ NPs

The TiO_2_ NPs synthesized by any route can be easily characterized by using UV-Vis spectrophotometry, Fourier transform infrared, X-ray diffraction, and electron microscopy for the confirmation of the formation of TiO_2_ NPs, elemental composition, and purity (Agarwal et al., 2022; Yadav et al., 2023b). Characterization of TiO_2_ NPs also becomes important to reveal the phase of the synthesized TiO_2_ NPs, as they may exist in three different forms under different physical and chemical conditions (Rathi and Jeice, 2023).

### 4.1. Characterization of TiO_2_ NPs by UV-Vis spectroscopy

In general, UV-Vis analysis of the TiO2 NPs is not relevant for the synthesis, but it is very important for calculating the band gap, especially when the synthesized TiO_2_ NPs are used in electronics (Abouhaswa, 2020). As a semiconductor, TiO_2_ NPs, in order to be used in electronics, must have an optimal band gap which can be calculated by UV-diffraction reflectance spectroscopy (UV-DRS). UV-Vis analysis provides a peak in the range of 300–400 nm depending on the morphology and route used for the synthesis of TiO_2_ NPs. Previously, several investigators have analyzed the bacterially synthesized TiO_2_ NPs by UV-Vis spectrophotometer and suggested the formation of TiO_2_ NPs by different types of bacteria, which is shown in Table 3.

**Table 3:** UV-Vis peaks obtained for the TiO_2_ NPs synthesized by different bacteria by different investigators.

**Bacteria used**	**Shape and size**	**Peaks (nm)**	**References**
*Halomonas elongata* IBRC-M 10214	Spherical (104.63 ± 27.75 nm)	300 and 400	Taran et al., 2018
*Bacillus subtilis*	Spherical and oval (66–77 nm)	366	Vishnu Kirthi et al., 2011
*Staphylococcus aureus*	Spherical and oval (20 nm)	324	Landage et al., 2020
*Propionibacterium jensenii*	Smooth, spherical (15–80 nm)	382 Band gap: 3.247 eV	Babitha and Korrapati, 2013
*Bacillus mycoides*	Spherical (40–60 nm)	381	Órdenes-Aenishanslins et al., 2014
*Bacillus subtilis FJ460362*	Spherical (10–30 nm)	379	Dhandapani et al., 2012
*Planomicrobium* sp.	Agglomerated, irregular shape (more than 100 nm)	400	Chelladurai et al., 2013
*Paenibacillus* sp. HD1PAH	Spherical (17.11 nm)	360	Chakravarty et al., 2023

From Table 3, it could be concluded that the UV-Vis peak for the TiO_2_ NPs synthesized by bacteria mainly falls above 350 nm and maximum to 400 nm wavelength, while in a few cases, the peak was also obtained below 350 nm. The variations in the peak for TiO_2_ NPs synthesized by bacteria could be mainly due to the presence of biologically different biomolecules, shapes, and sizes of the TiO_2_ NPs as size and morphology affect the surface plasmon resonance.

### 4.2. Characterization of TiO_2_ NPs by FTIR

When TiO_2_ NPs are synthesized by bacteria or any other microorganism, it becomes very important to know the biomolecules associated with the synthesized TiO_2_ NPs. FTIR analysis will reveal which functional groups are responsible for the stabilization and capping of the synthesized TiO_2_ NPs. Moreover, some of the organic compounds, such as glycyl-L-proline in the case of *A. hydrophila*, are responsible for the biotransformation of titanyl hydroxide into TiO_2_ NPs, which can be analyzed with FTIR in addition to other analytical tools. Jayaseelen et al. obtained bands in the range of 400–4,000 cm^−1^ for the bacterially synthesized TiO_2_ NPs. The major outcome from all the FTIR investigations was that the major and prominent bands were ~3,430, 2,937, 1,643, 1,403, 1,079 cm^−1^, and 700–500 cm^−1^. The bands of ~3,200–3,600 cm^−1^ could be attributed to the -OH stretching from an alcoholic group present in the enzymes or microbial proteins of the bacteria. The band at ~1,578 cm^−1^ indicates the presence of C–C ring stretching. The investigators reported that the bands at ~2,923 cm^−1^, 1,649 cm^−1^, and 679 cm^−1^ could be attributed to the lipids and proteins associated with the synthesis of TiO_2_ NPs (Landage et al., 2020). One major revelation made by FTIR by a group of investigators was that the band ~1,235 cm^−1^ indicates amide linkage between bacterial proteins and TiO_2_ NPs (Babitha and Korrapati, 2013), while the band for Ti-O stretching vibration could be obtained near 518 cm^−1^ (Chelladurai et al., 2013). Priyaragini et al. (2014) obtained four major bands for the TiO_2_ NPs synthesized by *S. bluensis* at 2,065.76 cm^−1^, 1,637.56 cm^−1^, 1,384.89 cm^−1^, and 644.22 cm^−1^ which were attributed to the C–H aldehyde stretching, C=C conjugate, NO_2_ conjugate, and alkynes, respectively.

Chakravarty et al. obtained the FTIR bands in the region of 3,400–2,400 cm^−1^ corresponding to the stretching vibration of terminating hydroxyl groups in samples, while the band at 1,350–1,000 cm^−1^ attributed to the *C*–N stretching of the amine group, and 1,550–1,350 cm^−1^ corresponds to nitro (N– – O) groups. In addition to this, the investigator also obtained intense bands at 500–700 cm^−1^ attributed to the Ti–O stretching band and Ti–O–Ti bridging stretching modes (Chakravarty et al., 2023).

### 4.3. Characterization of TiO_2_ NPs by XRD

Since TiO_2_ NPs exist in three different phases in nature, it becomes very important to analyze the synthesized TiO_2_ NPs by XRD. The different phases of TiO_2_ NPs are characterized by different peaks; for instance, a sharp peak near two theta 25–26° indicates the crystalline anatase phase, while the peak near two theta 27–28° is due to the rutile phase. Previously, numerous investigators synthesized TiO_2_ NPs by different bacteria under different temperatures, and other parameters are shown in Table 4.

**Table 4:** Major XRD peaks obtained by investigators earlier for TiO_2_ NPs synthesized by microorganisms.

**Peaks (two theta degrees)**	**Phase**	**Crystallite size (nm)**	**References**
25 28	Anatase (101) Rutile	–	Jha et al., 2009
23–24	Anatase crystalline (101)	–	Taran et al., 2018
27.811	Anatase crystalline (101)	–	Vishnu Kirthi et al., 2011
27.47	Rutile (110)	40.50	Jayaseelan et al., 2013
26	Anatase (101)	–	Landage et al., 2020
25.37 (101)	–	65	Babitha and Korrapati, 2013
25.37 (101)	–	–	Chelladurai et al., 2013
25.58 (101)	Anatase	15.23–87.6	Khan and Fulekar, 2016

### 4.4. Characterization of TiO_2_ NPs by electron microscopy

Electron microscopy (scanning and transmission) could be used to reveal the shape and size of the bacterially synthesized TiO_2_ NPs (Yang R. et al., 2022; Yang D. et al., 2023). The range of size becomes very important when it has to be applied in the fields of electronics and medicine. The electron microscopy could reveal the carbon molecules associated with the TiO_2_ NPs, as the non-metal area will be electron deficient and will appear darker in color in scanning electron microscopy, while in transmission electron microscopy that area will appear brighter in comparison with the dark Ti element. So, the data from electron microscopy in addition to FTIR and XRD could help in revealing the association of biomolecules with the TiO_2_ NPs (Liu et al., 2018; Zhang et al., 2023). Moreover, the elemental analyzer attached to the electron microscopy helps in revealing the chemical composition and purity of the synthesized TiO_2_ NPs. Various investigators have reported different sizes and shapes of the bacterially synthesized TiO_2_ NPs, which are already shown in Table 2. From the electron microscopic investigation of the previously reported study, it was found that in the majority of the cases, the bacterially synthesized TiO_2_ NPs were spherical in shape, while a few have also obtained an oval to irregular shape (Wang Z. et al., 2022; Xia et al., 2022). Some of them have also reported the aggregation of the TiO_2_ NPs synthesized by bacteria. The size of the synthesized TiO_2_ NPs by bacteria varied from 10 nm to above 100 nm, where the smallest size, i.e., 10–30 nm, was obtained by using *B. subtilis* by Vishnu Kirthi et al. (2011) and Dhandapani et al. (2012), and the largest size, i.e., 104.63 ± 27.75 nm, was obtained by Taran et al. (2018) by using *H. elongata* IBRC-M 10214.

## 5. Application of TiO_2_ NPs

Due to the unique properties of TiO_2_ NPs and their remarkable features, they are being used in every field of science, such as nanomedicine (Gupta et al., 2021), especially drug delivery wastewater treatment (Tang et al., 2021; Wang et al., 2021b), cosmetics, and food industries. Out of all these, TiO_2_ NPs are widely utilized in cosmetics and wastewater treatment. In this study, the applications of TiO_2_ NPs in various fields are described.

### 5.1. Application of TiO_2_ NPs in wastewater treatment

Wastewater is one of the foremost concerns nowadays. Sources of wastewater include industries, homes, factories, and transportation (Lito et al., 2012; Caprarescu et al., 2017). Water is an essential requirement for all living beings, so, due to the limited quantity of freshwater on the earth, it is of utmost importance to conserve water and recycle the wastewater (Chahar et al., 2023). The wastewater released by industries, domestics, factories, and transportation sources contains many contaminants such as heavy metals, toxic compounds, chemicals, and detrimental microorganisms (Yadav et al., 2020a). The pollutants inhaled by living beings will move from one tropic level to another, causing more absorption (Modi et al., 2022b; Yadav et al., 2023a). TiO_2_ NPs have shown the potential to eradicate all these contaminants much more efficiently. The photodegradation of the pollutants (dyes) present in wastewater is due to the photocatalytic effect of nanosized TiO_2_ (Agarwal et al., 2022). TiO_2_ NPs have been used in wastewater because they provide complete mineralization of pollutants (Panahi et al., 2018). The TiO_2_ NPs have been utilized in the laboratory as well as on-site for their property to clean wastewater. TiO_2_ helps to remove xenobiotic compounds from wastewater (Qamar and Ahmad, 2021). Photodegradation is a property of NPs that helps in the complete mineralization of organic pollutants without leaving behind any harmful by-products (Chenab et al., 2020).

TiO_2_ NPs have been proven to be effective in cleaning wastewater by reducing contaminants. The special antimicrobial activity makes TiO_2_ and other metal oxides suitable for the elimination of pathogenic microorganisms from wastewater. TiO_2_ NPs are able to produce ROS in a very short time, making them very effective for water treatment. TiO_2_ NPs remediate the toxic dyes from the wastewater by exhibiting a photocatalytic effect on the dyes in the presence of UV light (Pare et al., 2022).

Khan and Fulekar (2016) used TiO_2_ NPs synthesized by *B. amyloliquifaciens* for the removal of reactive red 31 in the presence of UV light. In this study, the investigators doped the TiO_2_ NPs with dopants such as Ag, Pt, La, and Zn and applied them for reactive red 31 dye removal. Furthermore, the authors concluded that Pt-doped TiO_2_ NPs were the most efficient in removing the dye at ~90.98%, while the as-synthesized TiO_2_ NPs removed the dye at only 75.83% (Khan and Fulekar, 2016). Table 5 shows the applications of TiO_2_ NPs and modified TiO_2_ nanocomposite in controlling the growth of pathogenic and non-pathogenic microorganisms.

**Table 5:** Remediation of water pollutants by using as-synthesized and modified TiO_2_ NPs/composites.

**TiO_2_ NPs and composites**	**Size (nm)**	**Dyes degraded**	**Complete inactivation time (min)**	**Removal percentage**	**References**
TiO_2_ NPs	22.11–97.28 (by TEM)	Reactive red 31		75%	Khan and Fulekar, 2016
Pt-doped TiO_2_ NPs				90.98%	
Ag-doped TiO_2_ NPs					
Zn-doped TiO_2_ NPs					
Ln-doped TiO_2_ NPs					
TiO_2_ NPs	2–18, spherical	MB		85.5%	Ngoepe et al., 2020
TiO_2_ NPs	24.19 ± 11.05	Rhodamine B		389.74 mg/g	Azeez et al., 2023
		Congo Red		244.57 mg/g	
Ag-TiO_2_/graphene aerogel (Ag–TiO_2_/GA–ATG)	–	Crystal violet		99.95%, pH 6.5, dye conc. 25 mg/L Catalyst dose: 29 mg	Trinh et al., 2023
		MB		97.11	
		Indigo Carmine		53.22%	
Ag/TiO_2_ nanoheteroparticles (ATNs)	5–100	RhB	90	TiO_2_ NPs alone: 69.8% (in sunlight) ATNs with 3 wt.% Ag: 90.1% ATNs with 8 wt.%: 88.7%	Shan et al., 2023
TiO_2_ NPs	Pseudo spherical shape 12.5	Indigo Carmine	70	–	Divya et al., 2022
N-doped TiO_2_ NPs (NT_3_M_4_)	6.3	Indigo Carmine (5 ppm)	70	99%	
Cellulose acetate CA@ TiO_2_ NPs (CTO)	16–72 (Avg:37.5)	MB (10 ppm), MR (30 ppm)	120	MB: ~25% MR: ~13% (Direct sunlight)	Mousa et al., 2021
TiO_2_ NPs (anatase)	Spherical, 12–18	MB	–	87%	Rathi and Jeice, 2023
TiO_2_ NPs	Spherical, 37.54	AR-79	60	84%	Priyaragini et al., 2014
		AR-80	60	85%	
Phyco-assisted TiO_2_ NPs	Cubic, spherical, ~50–90	MB	45	92.92%	Balaraman et al., 2022
*Aspergillus eucalypticola SLF1-assisted* TiO_2_ NPs	nanospheres	Reactive Blue 194	30	99.70%	Survase and Kanase, 2023

Priyaragini et al. photocatalytically degraded the two azo dyes (Acid Red 79 and Acid Red 80) by using crude extracts of *S. bluensis*, immobilized bacteria cells, and TiO_2_ NPs synthesized from them. The maximum degradation of both dyes with immobilized cells was 88% for AR-79 and 81% for AR-80, whereas with TiO_2_ NPs, AR-79, and AR-80 were found to be 84 and 85%, respectively. Moreover, investigators also remediated these dyes with crude extracts, with an efficiency of ~81% for AR-79 and 83% for AR-80. The investigators further conclude that free radicals of TiO_2_ NPs bind with the positively charged azo dyes and decolorize them. So, the maximum degradation of azo dyes was achieved with immobilized bacterial cells (Priyaragini et al., 2014).

Among all the investigations where TiO_2_ NPs and their nanocomposites were used for the remediation of pollutants from wastewater, the highest removal percentage of dye was noticed with crystal violet, which was 99.95% at 6.5 pH. In this study, the initial dye concentration was ~25 mg/L along with a catalyst dose of ~25 mg/L, and the photocatalyst dose was 29 mg. The nanocomposite used over here was Ag-TiO_2_/graphene aerogel (Ag–TiO_2_/GA–ATG). In one more attempt to photocatalytically degrade the Indigo Carmine dye by using nanocomposite nitrogen-doped TiO_2_ NPs [N-doped TiO_2_ NPs (NT_3_M_4_)], an efficiency of 99% was obtained within 70 min (Divya et al., 2022). As far as the dye removal efficiency of pure TiO_2_ NPs is concerned, the highest efficiency achieved was 87% for MB dye. In this study, the size of the synthesized anatase phase of TiO_2_ NPs was 12–18 nm, along with a spherical shape. There were only two attempts at the photocatalytic degradation of dye by using bacterial-mediated synthesized TiO_2_ NPs, one by Priyaragini et al. (2014) and another by Khan and Fulekar (2016). The size of the *B. amyloliquefaciens* mediates synthesized TiO_2_ NPs was 22.11–97.28 (by TEM), whose reactive red 31 dye degradation efficiency was 75% in comparison with the Pt-doped TiO_2_ NPs, whose efficiency was 90.98%. So, it could be concluded that TiO_2_ NPs alone cannot mineralize the dyes, so they must be used either in the nanocomposite form or in the metal-doped form for enhanced dye removal efficiency from the wastewater. When TiO_2_ NPs were used alone for dye removal, smaller-sized TiO_2_ NPs exhibited higher dye removal percentages; for instance, 87% of MB dye was removed with 12–18 nm-sized TiO_2_ NPs, whereas remediation of reactive red 31 dye was ~75% with TiO_2_ NPs of size 22.1–97.28 nm (Khan and Fulekar, 2016; Rathi and Jeice, 2023). Balaraman et al. (2022) attempted to degrade ~92.92% MB dye from wastewater by using 50–90 nm-sized TiO_2_ NPs from *S. myriocystum*.

### 5.2. Applications of TiO_2_ NPs for antimicrobial activity

TiO_2_ NPs not only find application in wastewater treatment, solar cells, and energy but also find application in the medical field, especially as an antimicrobial agent (Chen and Selloni, 2014). TiO_2_ NPs are also being used in drug delivery, where the nanosize helps them cross the blood–brain barrier, ultimately making them powerful against neurological disorders. TiO_2_ NPs themselves have antimicrobial properties, which makes them more useful in these areas. All the cells easily uptake the nanoparticle-based drugs, and they have very few side effects. Nanomedicines have been developed by using TiO_2_ NPs to be used in organ transplants, cosmetics treatments, and skin treatments (Sagadevan et al., 2022). The porous structure of titanium helps in the regeneration of bones and some muscles (Ouyang et al., 2019).

**Figure 7:**
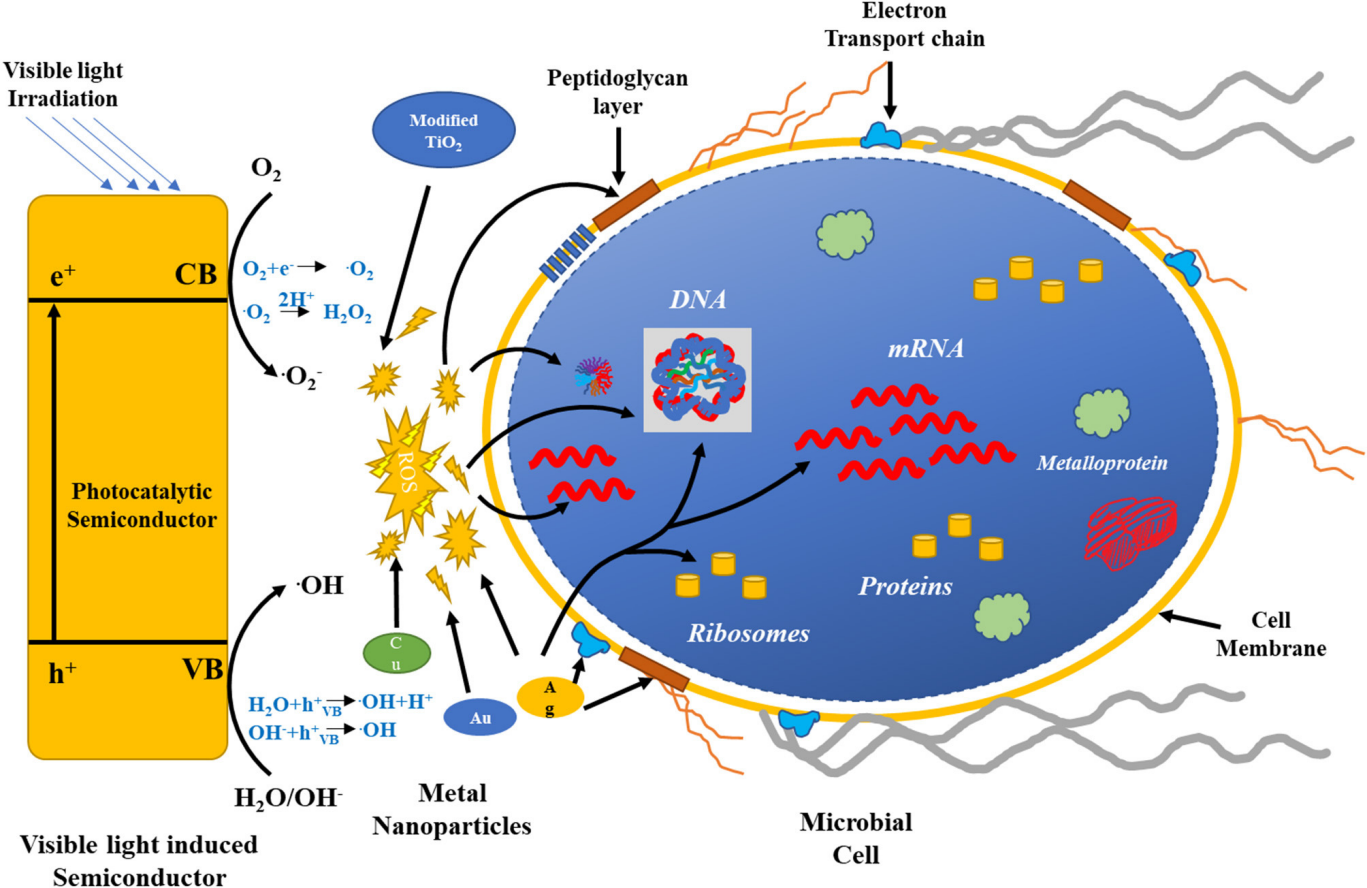
Possible mechanisms for the antimicrobial activity of the different metal oxide NPs and photocatalytic semiconductors.

Dental disinfectants based on the nanostructures of metal dioxide have been shown to be effective in relieving tooth- and gum-related problems. TiO_2_ NPs have been used in root canal treatment and as fillers because of their antibacterial properties, as reported by a group of investigators. Titanium and its oxides are used in dental care due to their non-corrosive properties (Jowkar et al., 2020; Raura et al., 2020; Liu et al., 2022). TiO_2_ NPs provide assistance in oral cancer therapy, but further research is needed to prove their functions *in vivo* (Sargazi et al., 2022). Figure 7 shows a possible phenomenon for the antimicrobial properties of the different metal oxide NPs and photocatalytic semiconductors (Regmi et al., 2018).

The left-hand side of the figure exhibits the activation of the photocatalytic semiconductor by visible light. ROS formation by various semiconductors destroys bacterial cell components, as shown by the red arrows. Ag, Cu, and Au NPs also generate ROS for a bactericidal effect. The green arrow represents targets of Ag. Reproduced with permission from Regmi et al. (2018).

Matsunaga et al. developed a Pt-loaded TiO_2_ (TiO_2_/Pt) and assessed its potential for antimicrobial and photoelectrochemical activities. The antibacterial effect of TiO_2_/Pt was assessed against *Lactobacillus acidophilus, S. cerevisiae*, and *E. coli* (Matsunaga et al., 1985). Another group of investigators exhibited that under UV irradiation, TiO_2_ NPs show photocatalytic antibacterial activity against viruses and multiple drug-resistant (MDR) bacteria (Bogdan et al., 2015). To date, several investigators have tried to increase the photocatalytic antibacterial properties of TiO_2_ NPs. To date, several attempts have been made for the development of visible light-responsive metal and non–metal-doped TiO_2_ NPs. Moreover, the antibacterial effect of these NPs was evaluated against Gram-negative bacteria such as *E. coli, Acinetobacter baumannii, Shigella flexneri*, and Gram-positive bacteria, for instance, *S. aureus, B. subtilis, Listeria monocytogenes*, and spores of *Bacillus anthracis* (Liou and Chang, 2012). This nano-TiO_2_ photocatalyst has been used for the eradication of pathogenic bacteria, thereby minimizing the spread of microbial-related illnesses. Markov and Vidaković (2014) performed the antimicrobial activity of TiO_2_ photocatalysts in a thin-film technique, petri-dish, and PTFE membrane-separated system. In this study, the investigators embedded the TiO_2_ NPs in the polymeric matrices in order to obtain nanocomposites (Charpentier et al., 2012; Hegedus et al., 2017). The major advantage of embedding the TiO_2_ NPs in the matrices was that there was no mechanical damage to the TiO_2_ NPs (Liao et al., 2013).

In addition to the antimicrobial activity/bactericidal activity of TiO_2_ NPs, they are also used as a photodynamic therapeutic (PDT) agent for killing cancerous cells in biomedical fields. It destroys cancer cells from the skin to the internal organs, both under UV and visible light sources (Liu et al., 2016). The PDT effect is exhibited due to the generation of ROS by TiO_2_. Moreover, such NPs can damage cellular respiration in mitochondria, which will release electron transfer proteins and eventually lead to cell death. When the TiO_2_ NPs are activated by the light, there is DNA fragmentation as a result of the electron transfer process. Such approaches exhibit the potential for reprograming gene coding either by deleting or inserting gene codons. Several investigators have also shown that the TiO_2_ nanotubes can be applied for the light-controlled delivery of drugs for the treatment of diseased tissues upon UV light illumination (Liu et al., 2016).

Ngoepe et al. reported the synthesis of TiO_2_ NPs by using *Monsonia burkeana* plant extract and used them for the photocatalytic degradation of MB dye and inhibition of *E. coli*. In this study, the author degraded the pollutants by ~85.5% of the simulated wastewater (Ngoepe et al., 2020). Table 6 shows the antimicrobial performance of TiO_2_ and modified TiO_2_ NPs under visible light.

**Table 6:** Antimicrobial performance of TiO_2_ and modified TiO_2_ NPs under visible light.

**Material**	**Size (nm)**	**Bacteria/fungi**	**Complete inactivation time**	**MBC (mm)**	**References**
TiO_2_ NPs	40.50, spherical	*Aeromonas hydrophila*			Jayaseelan et al., 2013
		*Escherichia coli*			
		*Pseudomonas aeruginosa*			
		*Staphylococcus aureus*		33 (ZOI)	
		*Streptococcus pyogenes*		31	
		*Enterococcus faecalis*			
(1–3 mol%) Ni/TiO_2_ NPs	8–10	*Escherichia coli*	>300 min (3% Ni dopant)		Yadav et al., 2014a
		*Staphylococcus aureus*	>240 min (3% Ni dopant)		
		*Salmonella abony*	>360 min (3% Ni dopant)		
(1–3 mol%) Cu/TiO_2_ NPs	9–10	*Escherichia coli*	240 min (3% Cu dopant)		Yadav et al., 2014b
		*Staphylococcus aureus*	120 min (3% Cu dopant)		
N/TiO_2_ NPs	10–30	*Escherichia coli*	420 min		Ananpattarachai et al., 2016
		*Staphylococcus aureus*	360 min		
0.5%Cu/TiO_2_ NPs	28.84	*Escherichia coli*	30 min		Mathew et al., 2018
		*Staphylococcus aureus*	30 min		
0.5 wt% MWNT/Fe-doped TiO_2_	Fe-doped TiO_2_: 15–20 MWNT diameter: 20–45	*Bacillus subtilis*	120 min		Koli et al., 2016a
		*Pseudomonas aeruginosa*	240 min		
(0.1–0.5%) MWNT/TiO_2_ NPs	TiO_2_ NPs: 8–15 MWNT diameter: 20–45	*Escherichia coli*	300 min (0.5% MWNT/TiO_2_)		Koli et al., 2016b
		*Staphylococcus aureus*	180 min (0.5% MWNT/TiO_2_)		
CS/Cu-doped TiO_2_	16	*Escherichia coli*	120 min		Raut et al., 2016
F-N-doped P25	70	*Escherichia coli*	60 min		Milosevic et al., 2017
Ag/TiO_2_ NPs	AgNPs: 0.9; TiO_2_ NPs: 8	*Escherichia coli*	60 min		Endo et al., 2018
(0.5%−2.5%) rGO/TiO_2_ NPs	17–18	*Escherichia coli*	75 min (1.5% rGO/TiO_2_ NPs)		Wanag et al., 2018
Cotton/(10%−50%) Mn-doped TiO_2_	Mn-doped TiO_2_: 150	*Staphylococcus aureus*	60 min (50 wt% Mn dopant)		Zahid et al., 2018
F-N-doped TiO_2_	21.3	*Escherichia coli*	60 min		Milošević et al., 2018
Cotton/(10–50%) Mn-doped TiO_2_	Mn-doped TiO_2_: 150	*Staphylococcus aureus*	90 min (25 wt% Mn dopant)		Zahid et al., 2018
		*Klebsiella pneumoniae*	90 min (25 wt% Mn dopant)		
TiO_2_ NPs (0.05 mg/ml)	2–18 nm (less dominant) 6–10 (more dominant)	*Escherichia coli*			Ngoepe et al., 2020
		*Staphylococcus aureus*	No activity		
TiO_2_ NPs	Spherical, 100	*Bacillus subtilis*			Rajeswari et al., 2023
		*Aspergillus niger*			
TiO_2_ NPs	10–30	*Staphylococcus aureus*			Al Masoudi et al., 2023
	–	*Bacillus subtilis*			
		*Escherichia coli*			
		*Klebsiella pneumoniae*			
		*Saccharomyces cerevisiae*			
		*Aspergillus niger*	20 μl/ml (MIC) and 40 μl/ml (MBC)		
		*Penicillium digitatum*			
TiO_2_ NPs	100	15 bacterial species: 10 clinical isolates and 5 environmental isolates (4 species of *Pseudomonas aeruginosa*, 4 species *of Staphylococcus aureus*, 3 of *Escherichia coli*, and 1 *Burkholderia cepacia, Enterobacter cloacae, Klebsiella oxytoca*, and Aeromonas)	–		Hazem Najem et al., 2023
Alpha-lipoic acid (ALA) functionalized bovine serum albumin (BSA) conjugate functionalized TiO_2_ NPs		Antibacterial and antifungal	–	–	Diana and Mathew, 2022
TiO_2_	Spherical 15–50 nm	*Escherichia coli*	Lowest MIC is 10.42 μg/ml		Thakur et al., 2019; Chen et al., 2020b; Wang H. et al., 2022
		*Bacillus subtilis*			
		*Salmonella typhi*	Lowest MIC is 10.42 μg/ml		
		*Klebsiella pneumoniae*	lowest MBC value, i.e., 83.3 μg/ml		
TiO_2_ NPs		*Candida albicans*	73% prevent the growth		Moradpoor et al., 2021
TiO_2_ NPs		*Candida parapsilosis*			Hifney et al., 2022
		*Prototheca ciferrii*			
TiO_2_ NPs		*Escherichia coli*			Trinh et al., 2023
		*Staphylococcus aureus*			
Ag/TiO_2_ nanohetero particles (ATNs)		*Staphylococcus aureus*	MIC: 250.00 mg/L	MBC: 250.00	Wang et al., 2021c; Shan et al., 2023
		*Enterococcus faecalis*	62.50	1,000.00	
		*Escherichia coli*	125.00	125.00	
		*Pseudomonas aeruginosa*	250.00	250.00	
		*Candida albicans*	MIC: 62.50 mg/L	1,000.00	
TiO_2_ NPs		*Staphylococcus aureus* (*MTCC-3160*)		At 800 g/ml, best effect	Divya et al., 2022; Nong et al., 2023; Wan et al., 2023
		*Aspergillus niger* (*MTCC-961*)		At 800 g/ml, best effect	
N-doped TiO_2_ NPs (NT3M4)		*Staphylococcus aureus*		21.6 mm (ZOI)	
		*Aspergillus niger*		10.2 mm (ZOI)	
Cellulose acetate CA@TiO_2_ NPs (CTO)		*Escherichia coli*		15 ± 0.8 mm	Mousa et al., 2021
TiO_2_NPs	Less than 50 nm	*Staphylococcus aureus*	MIC: 4.66 ± 0.20		Baig et al., 2020; Wan et al., 2023
		*Pseudomonas aeruginosa*	4.33 ± 0.19		
α-CuO@ TiO_2_		*Staphylococcus aureus*	2.33 ± 0.10		
		*Pseudomonas aeruginosa*	2.16 ± 0.09		
β-CuO@ TiO_2_		*Staphylococcus aureus*	2.00 ± 0.29		
		*Pseudomonas aeruginosa*	1.50 ± 0.29		
γ-CuO@ TiO_2_		*Staphylococcus aureus*	1.50 ± 0.14		
		*Pseudomonas aeruginosa*	1.08 ± 0.05		
Fe_3_O_4_@ TiO_2_/glycopolymers		*Escherichia coli*	Excellent in trapping *Escherichia coli*		Wang B. et al., 2021

From Table 6, it was found that either the TiO_2_ NPs or their nanocomposite were used against both Gram-positive and Gram-negative bacteria in addition to yeast such as *C. albicans* and *S. cerevisiae*. Out of the tested pathogens, Gram-negative bacteria *E. coli* were used most widely against the synthesized TiO_2_ NPs, followed by *Klebsiella pneumoniae, E. faecalis, and P. aeruginosa*. Among Gram-positive bacteria, *S. aureus* was extensively used for the evaluation of the synthesized TiO_2_ NPs (Yu et al., 2022; Wang et al., 2023). The antimicrobial activity of the TiO_2_ NPs synthesized by bacteria, along with their zone of inhibition (ZOI), is shown in Table 7.

**Table 7:** Antimicrobial activity of TiO_2_ NPs synthesized by bacteria, algae, and fungi against various pathogens along with their zone of inhibition.

**Tested microorganism**	**ZOI (mm)**	**Method used**	**References**
*Escherichia coli ATCC 25922* and *Staphylococcus aureus ATCC 43300*	No activity	Agar well diffusion	Taran et al., 2018
*Staphylococcus aureus*	33	Well diffusion and MIC	Jayaseelan et al., 2013
*Escherichia coli*	26		
*Aeromonas hydrophila*	23		
*Pseudomonas aeruginosa*	25		
*Streptococcus pyogenes*	31		
*Enterococcus faecalis*	29		
*Escherichia coli*	14	Disk diffusion	Landage et al., 2020
*Bacillus subtilis*	9	Disk diffusion	
*Bacillus subtilis* (3053)	9.6 ± 0.33 (50 μl), 0.1 ppm 13 ± 0.33 (100 μl), 0.2 ppm 17 ± 0.32 (200 μl), 0.3 ppm	Disk diffusion	Chelladurai et al., 2013
*Klebsiella planticola* (2727)	8 ± 0.33 (50 μl), 0.1 ppm 11 ± 0.33 (100 μl), 0.2 ppm 14 ± 0.33 (200 μl), 0.3 ppm	Disk diffusion	
*Aspergillus niger*	100–400 μl	Disk diffusion	
*Escherichia coli*	1 to 6 (20 μl) at 5 to 8 ppm		
*Staphylococcus aureus, S. epidermidis, Escherichia coli, Proteus vulgaris, Pseudomonas aeruginosa, and Klebsiella pneumoniae*			Balaraman et al., 2022
*Staphylococcus aureus, Escherichia coli, Pseudomonas aeruginosa, Klebsiella Pneumoniae and Bacillus subtilis*	40 μg ml^−1^ for *Escherichia coli*	Agar well diffusion	Rajakumar et al., 2012
–	–	Agar well diffusion	Survase and Kanase, 2023

From Table 7, it was found that the TiO_2_ NPs synthesized by bacteria and fungi were tested against Gram-positive, Gram-negative, and certain yeast. Among all the studies, it was found that the maximum ZOI obtained was 33 mm against *S. aureus*, while the minimum ZOI obtained was 9 mm against *B. subtilis*. The highest ZOI was obtained by the agar well diffusion method, while the lowest ZOI was obtained by the disk diffusion method. In addition to this, bacterially synthesized TiO_2_ NPs were also assessed against some of the common fungi, such as *A. niger*, which was inhibited at a concentration of 100–400 μl.

## 6. Conclusion

Titanium dioxide nanoparticles have gained huge attention in the last decade from investigators for photocatalytic material. Chemical approaches for the synthesis of TiO_2_ have restricted the applications of titanium dioxide in the biomedical field. Microorganisms, especially bacteria and fungi, are the preferred choice for the green synthesis of titanium dioxide nanoparticles due to their low growth time and eco-friendly nature. The presence of several enzymes and microbial proteins has played an important role in the biotransformation of titanium dioxide nanoparticles, in addition to the stabilization and capping agents. Quinones and oxidoreductases of microorganisms have been associated with the biosynthesis of titanium dioxide nanoparticles. The main mechanism for the formation of titanium dioxide nanoparticles is detoxification at the cellular level. The application of titanium dioxide has been used most widely as an antimicrobial agent due to its biocompatible nature. The antimicrobial activity of the titanium dioxide nanoparticles is associated with the formation of reactive oxygen species, which damage membrane lipids and denature proteins and DNA, leading to a release of the cytoplasmic content of the microorganism. The undoped pure titanium dioxide, doped one, and nanocomposite of titanium dioxide have shown tremendous potential for the photocatalytic degradation or mineralization of organic pollutants like dyes and pesticides. Some of the doped and titanium dioxide nanocomposites have shown complete mineralization of the organic pollutants under optimal conditions. So, such a green route-based approach for organic pollutant removal will open a new horizon in the field of nanophotocatalyst-based environmental cleanup.

## Author contributions

CR: Data curation, Investigation, Methodology, Writing—original draft, Writing—review and editing. VY: Data curation, Investigation, Methodology, Writing—original draft, Writing—review and editing. AG: Formal analysis, Investigation, Methodology, Software, Validation, Writing—review and editing. SA: Funding acquisition, Methodology, Resources, Software, Writing—review and editing. RV: Investigation, Methodology, Project administration, Supervision, Writing—review and editing. RC: Conceptualization, Data curation, Investigation, Visualization, Writing—review and editing. GG: Conceptualization, Data curation, Formal analysis, Visualization, Writing—review and editing. KY: Conceptualization, Formal analysis, Funding acquisition, Resources, Writing—review and editing. NC: Conceptualization, Formal analysis, Investigation, Methodology, Writing—review and editing. DS: Conceptualization, Methodology, Software, Supervision, Writing—review and editing. AP: Investigation, Methodology, Project administration, Visualization, Writing—review and editing.
